# High transcript abundance, RNA editing, and small RNAs in intergenic regions within the massive mitochondrial genome of the angiosperm *Silene noctiflora*

**DOI:** 10.1186/s12864-015-2155-3

**Published:** 2015-11-14

**Authors:** Zhiqiang Wu, James D. Stone, Helena Štorchová, Daniel B. Sloan

**Affiliations:** Department of Biology, Colorado State University, Fort Collins, CO 80523 USA; Institute of Experimental Botany v.v.i, Czech Academy of Sciences, Prague, Lysolaje 16502 Czech Republic

**Keywords:** Antisense RNA, Junk DNA, mtDNA, ORFs, Plant mitochondrial genome, Regulatory RNA, Spurious transcription

## Abstract

**Background:**

Species within the angiosperm genus *Silene* contain the largest mitochondrial genomes ever identified. The enormity of these genomes (up to 11 Mb in size) appears to be the result of increased non-coding DNA, which represents >99 % of the genome content. These genomes are also fragmented into dozens of circular-mapping chromosomes, some of which contain no identifiable genes, raising questions about if and how these ‘empty’ chromosomes are maintained by selection. To assess the possibility that they contain novel and unannotated functional elements, we have performed RNA-seq to analyze the mitochondrial transcriptome of *Silene noctiflora*.

**Results:**

We identified regions of high transcript abundance in almost every chromosome in the mitochondrial genome including those that lack any annotated genes. In some cases, these transcribed regions exhibited higher expression levels than some core mitochondrial protein-coding genes. We also identified RNA editing sites throughout the genome, including 97 sites that were outside of protein-coding gene sequences and found in pseudogenes, introns, UTRs, and transcribed intergenic regions. Unlike in protein-coding sequences, however, most of these RNA editing sites were only edited at intermediate frequencies. Finally, analysis of mitochondrial small RNAs indicated that most were likely degradation products from longer transcripts, but we did identify candidates for functional small RNAs that mapped to intergenic regions and were not associated with longer RNA transcripts.

**Conclusions:**

Our findings demonstrate transcriptional activity in many localized regions within the extensive intergenic sequence content in the *S. noctiflora* mitochondrial genome, supporting the possibility that the genome contains previously unidentified functional elements. However, transcription by itself is not proof of functional importance, and we discuss evidence that some of the observed transcription and post-transcriptional modifications are non-adaptive. Therefore, further investigations are required to determine whether any of the identified transcribed regions have played a functional role in the proliferation and maintenance of the enormous non-coding regions in *Silene* mitochondrial genomes.

**Electronic supplementary material:**

The online version of this article (doi:10.1186/s12864-015-2155-3) contains supplementary material, which is available to authorized users.

## Background

The longstanding debate about the meaning and existence of “junk DNA” has come under renewed scrutiny in light of recent and controversial claims that 80 % of the human genome is functional [1–5]. While most of this debate has focused on the nucleus, the evolution of non-coding sequence content in mitochondrial genomes has also been of great interest, particularly in plants in which mitochondrial genomes are large and variable in size relative to other eukaryotes and have low gene densities [6–12]. Angiosperm mitochondrial genomes are typically a few hundred kb in size, but they range anywhere from 66 to 11,318 kb [9, 10]. This genome size variation largely reflects differences in the amount of non-coding content, which comes from diverse sources including repeats and large duplications [13–15], intracellular gene transfer (IGT) of nuclear and plastid DNA [16–18], and horizontal gene transfer (HGT) from other species [19–21]. In addition, a large proportion of intergenic content is of unidentifiable origins and is not conserved across related angiosperm species [12].

The origin and maintenance of mitochondrial non-coding content is particularly intriguing in the angiosperm genus *Silene* (Caryophyllaceae). Some species in this genus have massive mitochondrial genomes (7–11 Mb), in which more than 99 % of the genome content is identified as intergenic sequence (IGS) [9, 22]. These mitochondrial genomes also have an unusual multichromosomal structure, in which the genome is fragmented into dozens of circular-mapping chromosomes. Surprisingly, many of these chromosomes appear to be ‘empty’, lacking any identifiable genes or functional elements. Similar phenomena have also been reported in two other angiosperm species: *Cucumis sativus* [15] and *Amborella trichopoda* [20]. These observations raise basic questions about how and why such empty chromosomes are faithfully maintained, replicated and transmitted. One possibility is that they are not. We recently found that different populations of *S. noctiflora* varied in the presence or absence of entire chromosomes, suggesting that there may be an ongoing process of chromosome loss [22]. However, we also found that many of the seemingly empty chromosomes are shared across populations, raising the possibility that they are conserved by natural selection and contain some type of unidentified functional elements.

Previous research examining transcriptional patterns in animal mitochondrial and plant plastid genomes has identified non-coding RNAs and small RNAs from IGSs that potentially play a regulatory role in the control of gene expression [23–28]. Also, the existence of novel protein-coding genes has been observed in the mitochondrial genomes of plants and other eukaryotes, including chimeric open reading frames (ORFs) responsible for cytoplasmic male sterility (CMS) in angiosperms [29], a homolog of the bacterial *mutS* gene in octocorals [30–32], and ORFs associated with doubly uniparental inheritance in bivalve molluscs [33, 34].

Genome-wide patterns of transcription in plant mitochondria are only beginning to be explored [35], but high-throughput cDNA sequencing (RNA-seq) has been successfully used to compare gene expression across developmental stages and tissues [36], identify transcribed ORFs and IGSs [37, 38], and detect post-transcriptional processing and RNA editing in both coding and non-coding regions [38–40]. Mitochondrial transcriptome analysis poses some unique challenges in plants. In particular, the presence of insertions from the plastid and nuclear genomes can make it difficult to assess whether transcripts matching these regions are actually originating from the mitochondrial genome. This is particularly relevant in light of the recent observation that the mitochondrial genomes of *Arabidopsis thaliana* and related species in the Brassicaceae contain a nuclear-derived gene that is actively transcribed [41]. Purifying or enriching for mitochondrial RNA and making comparisons to total cellular RNA are one approach to help identify true mitochondrial transcription in these cases.

Here, we use RNA-seq to examine patterns of transcript abundance and RNA editing in the 7-Mb mitochondrial genome of *S. noctiflora* in order to identify candidate functional elements that could potentially explain the maintenance of enormous quantities of non-coding content and the existence of seemingly empty chromosomes in this genome.

## Results

### Illumina sequencing, read mapping, and library validation

We generated Illumina RNA-seq data for both total cellular and mitochondrial-enriched samples from *Silene noctiflora* leaf tissue (each with two biological replicates). Each RNA sample was used for both small RNA-seq (transcripts <30 nt) and more conventional RNA-seq based on fragmentation of full-length transcripts. For convenience, we will refer to the latter as mRNA-seq throughout even though that dataset also contains reads corresponding to non-coding and structural RNAs. Each mRNA-seq library produced between 35.9 and 44.6 million reads, while the small RNA libraries each produced between 13.7 and 18.7 million reads (Table 1). After trimming and quality/length filtering, an average of 34.8 % mRNA-seq reads from the two replicated mitochondrial-enriched libraries could be mapped to the reference mitochondrial genome. In contrast, less than 5 % of the reads from the total cellular libraries mapped to the mitochondrial reference, confirming the effectiveness of our mitochondrial-enrichment procedure. Analyzing individual protein-coding, rRNA, and tRNA genes also revealed strong and consistent enrichment in the mitochondrial libraries (Additional file 1: Table S1). The small RNA-seq libraries showed similar evidence of mitochondrial enrichment, but the overall proportion of small RNA-seq reads mapping to the mitochondrial reference genome (an average of 2.6 % and 0.5 % for mitochondrial-enriched and total cellular libraries, respectively) was much smaller than observed for the mRNA-seq libraries (Table 1).

**Table 1:** RNA-seq library statistics

Library	Sample	Replicate	Raw reads	Filtered reads	Mapped reads	Mapping rate
mRNA-seq	Mitochondrial	1	41,487,281	40,795,778	14,001,209	34.32 %
	Mitochondrial	2	40,959,060	40,287,889	14,232,280	35.33 %
	Total Cellular	1	35,895,157	35,230,681	1,572,554	4.46 %
	Total Cellular	2	44,610,884	43,739,586	1,851,351	4.23 %
Small RNA-seq	Mitochondrial	1	13,747,462	9,185,676	218,560	2.38 %
	Mitochondrial	2	17,065,766	9,695,677	264,224	2.73 %
	Total Cellular	1	18,703,819	10,518,853	46,361	0.44 %
	Total Cellular	2	17,889,739	9,941,996	49,189	0.49 %

Estimates of mitochondrial transcript abundance were highly consistent between pairs of biological replicates (Additional file 1: Figure S1) for both mRNA-seq libraries (*r* > 0.94) and small RNA-seq libraries (*r* > 0.82). However, there was a conspicuous difference between the two biological replicates for the mitochondrial-enriched mRNA-seq libraries, with a set of highly expressed sequences that were overrepresented in replicate 2 (Additional file 1: Figure S1). Manual inspection revealed that these sequences were all found in mitochondrial rRNA genes, indicating that rRNA depletion was less effective for that replicate.

The read mapping results also conformed to basic biological expectations. Within annotated genes, there was an overwhelming bias towards transcripts originating from the coding strand (Additional file 1: Figure S2), confirming the effectiveness of the strand-specific library construction and analysis. There was also a major reduction in transcript abundance for introns relative to annotated exons (although substantial levels of intronic transcripts were still detectable; Additional file 1: Figure S2b).

### mRNA-seq analysis of genic and intergenic mitochondrial transcripts

#### Protein-coding genes

**Fig. 1:**
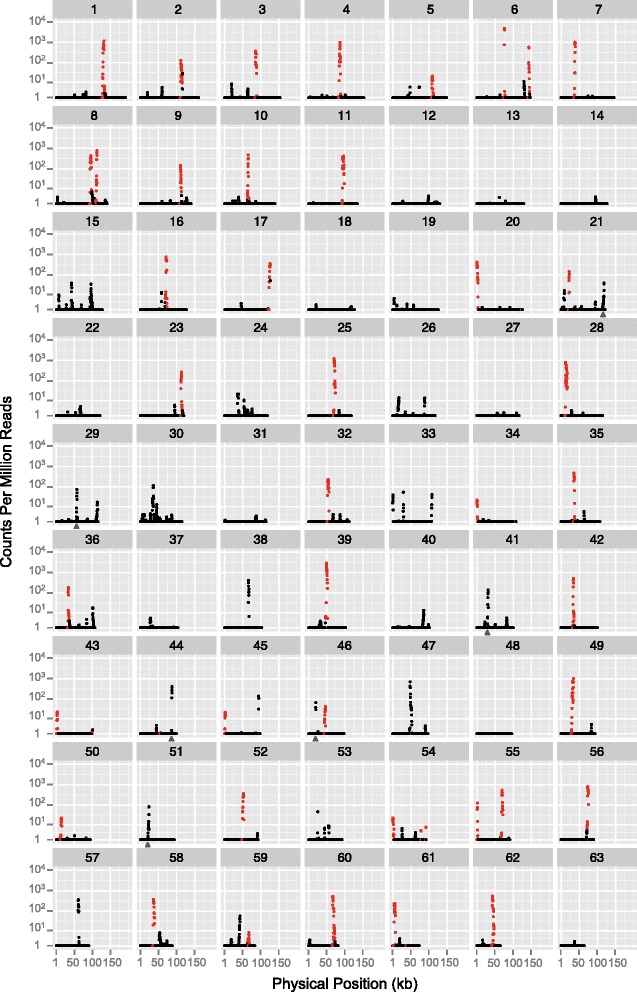
mRNA-seq read depth across the 63 chromosomes of the *Silene noctiflora* mitochondrial genome. Coverage estimates (in counts per million reads) are based on a sliding window with a window size of 500 bp and a step size of 250 bp for the average of two mitochondrial-enriched libraries. Coverage values for forward and reverse strands were combined. The red points represent annotated gene regions, including introns and 2 kb of 5’ and 3’ flanking sequences, whereas the black points represent sequences outside of these regions. The gray triangles indicate high-depth regions with sequence identity with intact mitochondrial genes from elsewhere in the genome such that read depth estimates may be the results of cross-mapping. The plot was generated with the ggplot2 library (http://ggplot2.org/) in R

We found high transcript abundances (>1000× coverage in the mitochondrial-enriched libraries) for all annotated protein-coding genes in the *S. noctiflora* mitochondrial genome (Fig. 1; Additional file 1: Tables S1 and S2). Average read depths spanned more than two orders of magnitude, with the lowest read depth for *ccmFn* (1311×) and the highest for *atp9* (359,636×). There was a high level of read coverage across the entire coding-sequence of each gene with the exceptions of *nad6* and *ccmC*, which both exhibited extreme drops in coverage before the first in-frame stop codon (Additional file 1: Figure S3) as has been observed in other angiosperms [42–44]. For the majority of the remaining protein-coding genes (17 of 24), there was a sharp drop-off in coverage within 100 nt after the annotated stop codon (Additional file 1: Figures S2 and S3), indicating efficient 3′ transcript processing, which is often mediated by t-elements [43, 45, 46].

#### *Pseudogenes*

**Fig. 2:**
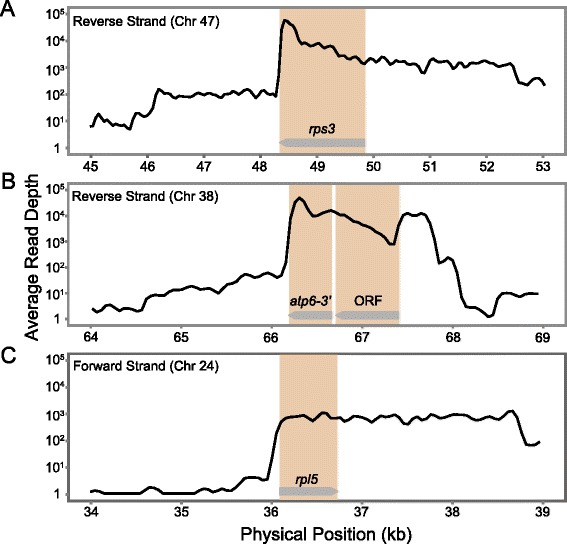
mRNA-seq read depth in some of the chromosomal regions containing annotated pseudogenes. Coverage estimates (in terms of average read depth) are based on a sliding window with a window size of 100 bp and a step size 50 bp for the average of two replicates of mitochondrial-enriched libraries. Coverage values are only reported for the coding strand. **a** the annotated *rps3* pseudogene on chromosome 47; (**b**) the duplicated copy of the 3’-end of *atp6* preceded by a 729-bp ORF on chromosome 38 (note that this region is also duplicated on chromosome 57); (**c**) the annotated *rpl5* pseudogene on chromosome 24 containing internal nonsense and frame-shift mutations (Additional file 1: Figure S4). The brown shading indicates the boundaries of the pseudogenes and the ORF, and the gray arrows indicate the orientation of the coding strand

We detected expression of multiple annotated pseudogenes [9, 22]. In particular, there were three lines of evidence that *rps3*, which was previously annotated as a pseudogene because of a truncated 5′ end, is actually functional as a protein-coding sequence. First, it was expressed at high levels that are well within the range observed for other protein-coding genes (Fig. 2a). Second, it exhibited a steep drop-off in coverage right after its stop codon suggesting active processing of 3’ ends (Fig. 2a). Third, it contained multiple sites that underwent cytidine-to-uridine (C-to-U) RNA editing at high frequency (see below; [47]). Because *rps3* is not located near any other annotated gene, it is unlikely that the high levels of expression were caused by co-transcription or run-on transcription.

We also found relatively high levels of expression of a duplicated copy of *rpl5* that shares 90 % nucleotide sequence identity with the annotated functional copy but is interrupted by internal nonsense and frame-shift mutations (Fig. 2c; Additional file 1: Figure S4). In this case, however, coverage levels were below what was observed for most other protein-coding genes, and there was no evidence of 3’ processing (Fig. 2c), so this gene may be transcribed as a byproduct of its similarity to the intact *rpl5* copy. In addition, there was high transcript abundance in a large region containing a perfect duplication of a 390-bp region from the 3’ end of *atp6* (Fig. 2b). This region also contained the largest transcribed ORF of unidentified function in the genome (see below; Additional file 1: Table S3). Finally, there were some examples of annotated pseudogenes that appear to be relatively recent duplicates of other intact genes in the genome and contain internal regions that share 100 % identity with their functional counterparts (e.g., *mttB* and *nad2*). In these cases, coverage estimates most likely represented duplicate mappings based on transcripts that originated from the intact copy of the gene (Additional file 1: Figure S5).

#### Intergenic regions and ‘empty’ chromosomes

Most regions outside of annotated genes and pseudogenes showed little or no evidence of transcription (Fig. 1). However, we did identify a large number of intergenic (i.e., unannotated) regions with localized areas of high transcript abundance, often reaching levels observed for known protein-coding genes (Fig. 1). Many of these regions were found on the empty chromosomes that lack any annotated functional element. In fact, only two chromosomes (27 and 48) did not contain any regions with an average transcript abundance of >100× (which was chosen as a threshold because it represented the ~5 % tail of the distribution of coverage across the genome; Additional file 1: Figure S6). The vast majority of expressed IGSs were overrepresented in the mitochondrial-enriched mRNA-seq libraries (Additional file 1: Figure S7), suggesting that the corresponding transcripts exist within the mitochondria and are transcribed from the mitochondrial genome. There were only two regions that had average read depths of >100× and were overrepresented in total cellular mRNA-seq libraries. These corresponded to the transferred fragments of the plastid gene *psaA* (chromosome 21) and the nuclear (cytosolic) 26S rRNA gene (chromosome 53), indicating that the expression estimates for these two regions of the mitochondrial genome were the result of cross-mapping of transcripts that actually originated from other genomes.

#### ORFs

Many of the unannotated regions with high levels of transcript abundance contained ORFs. In total, we identified 7339 ORFs that were located more than 2 kb from any annotated genes and had a minimum length of 201 bp. Of these, 65 had an average read depth of >100× (Additional file 1: Table S3), raising the possibility that they function as novel protein-coding sequences. However, after excluding ORFs that are duplicated portions of known mitochondrial protein-coding genes, nearly all of these sequences are very short (<400 bp) and lacked detectable similarity with any sequences in the NCBI nr/nt databases (Additional file 1: Table S3), making it difficult to assess whether they have any functional importance. Many ORFs in this size range would be expected at random given the large amount of sequence in the *S. noctiflora* mitochondrial genome (7 Mb), and none of the ORFs exhibited detectable similarity to known sequences other than the standard set of mitochondrial and plastid protein genes (Additional file 1: Table S3). One notable outlier was a 729-bp ORF that occurs in a highly expressed region (Fig. 2b) and is just upstream of a duplicated fragment from the 3’ end of *atp6* (Fig. 2b). The ORF itself also shares a short region of sequence similarity (72 amino acids with 57 % identity) with a 5’ extension in the annotated *atp6* gene in the mitochondrial genome of *Hyoscyamus niger* [48]. It also contains a predicted transmembrane domain near the C-terminal end of the translated protein sequence (amino acid positions 191–215). These observations make the 729-bp ORF the most promising candidate for a novel protein-coding gene in the *S. noctiflora* mitochondrial genome. Interestingly, this ORF is present in two identical copies as part of larger duplication shared between chromosomes 38 and 57, but it is absent entirely from the sequenced mitochondrial genome from a different population of *S. noctiflora* [9].

### Mitochondrial small RNA-seq

**Fig. 3:**
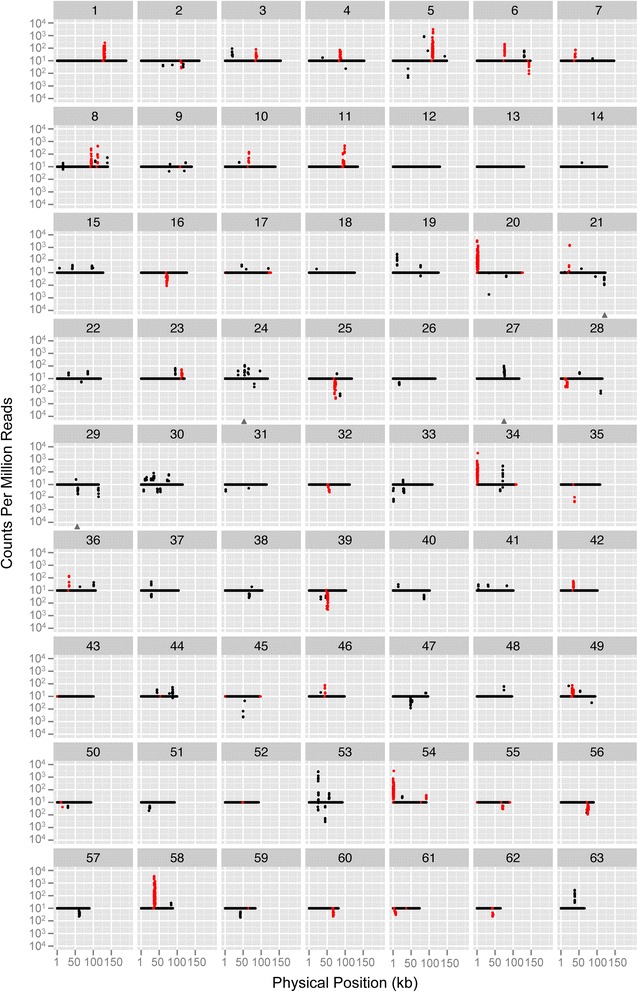
Small RNA-seq read depth across the 63 chromosomes of the *Silene noctiflora* mitochondrial genome. Coverage estimates (in counts per million reads) are based on a sliding window with a window size of 50 bp and a step size of 25 bp for the average of two mitochondrial-enriched libraries. Coverage values for forward and reverse strands were displayed above and below the x-axis, respectively. The red points represent annotated gene regions including introns and 2 kb of 5’ and 3’ flanking sequences, whereas the black points represent sequences outside of these regions. The gray triangles indicate high-depth regions with sequence identity with intact mitochondrial genes from elsewhere in the genome such that read depth estimates may be the results of cross-mapping. The plot was generated with ggplot2 library in R

**Fig. 4:**
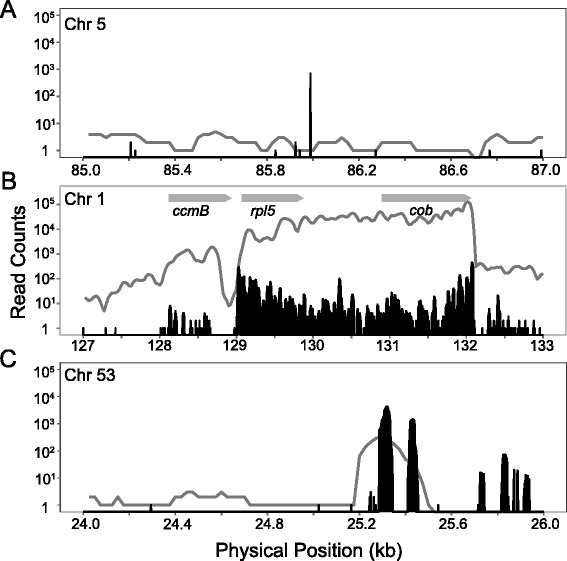
Examples of regions with small-RNA expression. The vertical black lines represent position-specific read counts for small RNAs averaged from two mitochondrial-enriched libraries. The gray lines represent mRNA-seq coverage based on a sliding window as described for Fig. 1. Coverage estimates are based only on reads mapping to the forward strand. **a** an example of a small RNA with reads all mapping to the same start site in a region that does not contain longer RNA transcripts; (**b**) an example of a region with highly expressed protein-coding genes and abundant small RNAs that are likely derived from degraded mRNA transcripts; gray arrows indicate the orientation of the coding strand; (**c**) an example of an IGS with both small RNAs and longer transcripts; note that the small RNAs are not localized to a single start site

The overall frequency of small RNA reads (17–25 nt) that mapped to the mitochondrial genome was very low (Table 1), and the largest populations of these reads were from the coding (sense) strand of annotated mitochondrial genes (Fig. 3). In general, these reads were overlapping and spread widely across the genic regions (Fig. 4b), suggesting that they represented degradation products from longer mRNA and rRNA transcripts rather specific functional classes of small RNAs. We did not find any candidates for antisense regulatory RNAs (i.e., highly expressed and antisense to annotated mitochondrial genes).

In addition to the large fraction of mapped reads associated with protein-coding and rRNA genes, there were many small RNA-seq reads that mapped outside of regions containing annotated genes (Fig. 3). For most of these regions, reads were overrepresented in mitochondrial-enriched libraries (Additional file 1: Figure S8), suggesting that many of the identified small RNAs are localized to the mitochondria and likely originated from the mitochondrial genome. Overall, there was a positive correlation between small RNA abundance and read depth from mRNA-seq libraries (*r* = 0.597; Additional file 1: Figure S9), further indicating that many of the intergenic small RNAs may be derived from longer intergenic transcripts (Fig. 4c). However, we also found examples of small RNAs in regions of the mitochondrial genome with little or no evidence of expression in the mRNA-seq libraries (Fig. 4a). We considered large sets of reads that shared the same start site and length with few or no overlapping reads at neighboring sites to be the strongest candidates for functional small RNAs but found only nine such examples with a read depth of >50× (Table 2). Of these, only four showed evidence of overrepresentation in the mitochondrial-enriched libraries, and none of them exhibited significant similarity to characterized small RNAs in miRBase.

**Table 2:** Identified small RNAs. Mito and TC refer to the mitochondrial-enriched and total-cellular libraries, respectively

Chrom	Start	End	Length (bp)	Strand	Reads counts				
					Mito 1	Mito 2	TC 1	TC 2	Sequence
5	41506	41527	22	-	200	235	227	215	CAATTCCTTTGAGTTTCATTCT
5	85989	86005	17	+	688	750	23	14	GTGGCTGGATTGAATCC
8	138948	138969	22	+	42	101	9	4	ACAAGGAGGAAAAGATCTATCT
20	33127	33145	19	-	561	622	822	495	AGAGGTGAAATAGAATAAC
25	85974	85990	17	-	157	247	550	517	GTTTTCATGAGGCTGAT
28	110134	110152	19	-	133	122	206	215	CCTCTCAAGTCATTTCACA
45	49949	49971	23	-	483	411	76	60	CGGCACGAGCTGACGACAGCCAT
48	74534	74551	18	+	48	69	194	366	GAATCCGGGCCAGAAGCG
49	21331	21347	17	+	55	63	20	24	AGTCAGAATCCGGGCCA

### RNA editing in protein-coding and non-coding sequences

Post-transcriptional C-to-U editing of RNA transcripts is common in plant mitochondrial genomes [49]. By comparing mapped mRNA-seq reads to the *S. noctiflora* reference mitochondrial genome, we identified a total of 290 RNA editing sites (Additional file 1: Tables S4 and S5) with a minimum read depth of 100× and a minimum of 20 % edited reads (not counting duplicate sites in large repeat regions). Approximately 96 % (279) of these sites exhibited the expected pattern of C-to-U editing (Tables 3 and Additional file 1: Table S4). Eleven unique non-canonical (i.e., non-C-to-U) editing sites were also predicted (Additional file 1: Table S5), three of which were duplicated and found in both identical copies of a repeat shared by chromosomes 21 and 41. Inspection of the original reference genome assembly revealed that there were actually slight variations at the genomic level between these repeats in chromosomes 21 and 41 that were overlooked during the assembly process. Therefore, these three apparent RNA editing sites are actually the result of errors in the reference genome. Manual inspection of the remaining eight non-canonical editing sites revealed that they were dependent on mismatches at the very ends of Illumina reads. Based on this observation, we repeated read mapping and variant calling after trimming an additional five bp from the end of each read. None of the eight sites were identified as being edited in this re-analysis. For four of the eight sites, the spurious editing predictions were consistent with the presence of one bp of Illumina adapter sequence at the 3’ end of some reads (which was too short to be recognized as adaptor sequence during the read trimming process). The other four sites could not be explained by the presence of adapter sequence, but they were based on reads containing multiple mismatches of different types at their 3’ ends, so it is very unlikely that they are true RNA editing sites. In contrast to the non-canonical editing sites, none of the predicted C-to-U editing sites were affected by the re-analysis after additional end trimming (data not shown).

**Table 3:** Summary of identified C-to-U RNA editing sites. Values shown in parentheses indicate the number of RNA editing sites after excluding duplicate genes. Editing frequency values were calculated after excluding duplicates. UTRs and IGSs were defined/distinguished based on an arbitrary cutoff of 2 kb from annotated coding sequences

	Editing sites	Mean editing frequency (%)
Protein-Coding	228 (182)	91.1
Synonymous	23 (17)	60.6
Nonsynonymous	205 (165)	94.2
Pseudogene	30 (24)	85.7
Intron	14 (12)	59.8
UTR	36 (32)	54.7
IGS	29 (29)	62.3
Total	337 (279)	82.1

The set of 182 (unique) editing sites identified within protein-coding sequence was similar to the set of 189 sites reported in a previous analysis of RNA editing in another population of *S. noctiflora* [47], with the two sets sharing 175 sites in common (Additional file 1: Table S6). All seven of the newly identified sites in this study were only partially edited at low frequencies with a maximum of 42 % edited reads. Of the 14 sites that were identified previously but not in the present study, nine were found in *ccmB*. Oddly, manual inspection of read mapping found that eight of the nine were, in fact, edited at a high frequency of >80 % (Additional file 1: Table S7). Another of the missing sites (in the first exon of *nad1*) was also found to be edited at a frequency of >80 %, and two other sites (in *mttB* and the fourth exon of *nad2*) were found to exhibit evidence of editing albeit at a frequency below the 20 % cutoff applied in our study (Additional file 1: Table S7). Therefore, only two of the previously identified sites (one in *ccmB* and another in *ccmFn*) appeared to be truly unedited in our dataset.

The reason why some sites with evidence of high frequencies of editing in our dataset were not identified by the variant detection analysis in CLC appears to be related to the fact that the corresponding genes are part of large identical repeats found on multiple different chromosomes. By repeating read mapping and variant calling after eliminating chromosomes with duplicate copies of *ccmB* from the reference genome, we found that all sites were successfully identified. Therefore, it appears that he CLC variant caller behaves erratically when there are large repeats in the reference genome, failing to identify some (but not all) variants in these regions, which raises the possibility that some RNA editing sites in other parts of the genome (particularly large repeated regions) were missed in our analysis.

Our analysis identified dozens of editing sites in introns, untranslated regions (UTRs), and annotated pseudogenes, as well as many of the novel IGS transcripts discovered in this study (Table 3). However, most of these sites were only partially edited, with introns, UTRs, and IGSs all exhibiting average editing frequencies of 62 % or lower in contrast to an average editing frequency of 94 % for nonsynonymous sites in protein-coding genes (Table 3).

## Discussion

We have performed a genome-wide analysis of mitochondrial transcription in *Silene noctiflora* to assess the possibility that there are uncharacterized functional elements that could explain the maintenance of the massive quantities of intergenic DNA and empty mitochondrial chromosomes in this species. We found multiple lines of evidence that support the possibility that IGSs in *S. noctiflora* contain novel functional elements: 1) there were numerous regions with high levels of transcript abundance that reached levels observed for some of the major mitochondrial OXPHOS genes; 2) many of the highly transcribed regions contained ORFs that could potentially function as protein-coding sequence; 3) there were identifiable small RNAs that mapped with high depth to specific locations in the mitochondrial genome and were overrepresented in the mitochondrial-enriched libraries; 4) many of the transcribed IGSs were subject to C-to-U RNA editing.

However, an important lesson (or reminder) from the recent debate over the ENCODE project is that transcription is not necessarily an indication of functional importance [2–5]. Given the low information-content of promoter elements, it is expected that some “spurious‘ transcription would occur even in random DNA sequence [50–53]. Therefore, each of the above observations should be interpreted cautiously. Indeed, there are some reasons to believe that many of the identified transcribed elements are not functionally important or conserved by selection. First, the short lengths of the identified ORFs and their general lack of detectable homology or reading-frame conservation with related species make it difficult to infer functionality [37, 38]. Second, with respect to small RNAs, the list of candidates was very short, and the overall mapping rate for small RNAs was much lower than for longer transcripts (Tables 1 and 2). Third, even though RNA editing in plant mitochondria generally performs an important functional role by restoring conserved sequences [54], it is likely that there is also some non-adaptive “misfiring” of the editing machinery, which may explain why most editing observed at synonymous sites is only partial (Table 3) [47, 55, 56]. Partial editing was also the norm for sites that were identified in intergenic transcripts in *S. noctiflora* leaf tissue (Table 3). Therefore, it is possible that the observed editing of intergenic transcripts is not indicative of their functional importance, but rather a byproduct of enzymatic machinery that has evolved to target hundreds of functionally important sites in protein-coding genes and occasionally edits off-target sites.

If the novel transcribed regions identified in this study are functionally important and responsible for the maintenance of empty chromosomes in the *S. noctiflora* mitochondrial genome, we would predict that chromosomes containing these elements are more likely to be conserved among *S. noctiflora* populations. We previously showed that, of the 23 empty chromosomes in the mitochondrial genome of the population used in this study (BRP), 15 were shared with a second population of *S. noctiflora* (OSR), whereas eight were unique to BRP. Only two (25 %) of the unique chromosomes contained at least one transcribed region with an average read depth >1000×, and in both cases this was the same 729-bp ORF that is duplicated on chromosomes 38 and 57. In comparison, six of the 15 shared chromosomes (40 %) contained such highly expressed regions. Thus, there is a weak trend supporting the prediction that having highly expressed regions is a predictor of conservation for these empty chromosomes. However, given that the presence of at least some transcriptional activity (i.e., >100× read depth) was almost ubiquitous for the entire set of chromosomes in the genome (Fig. 1), it is difficult to see any clear distinction between the shared and unique chromosomes.

Because the *S. noctiflora* mitochondrial genome is so large and unusual in structure, it is of interest to consider how the observed patterns of intergenic expression compare to previous studies examining mitochondrial transcripts in angiosperms with more conventional mitochondrial genomes. Some of our key observations about transcription and RNA editing in IGSs have also been found in species with much smaller mitochondrial genomes. First, intergenic regions that are transcribed at rates similar to those of core mitochondrial genes have been identified in both monocots and eudicots [38, 52, 57, 58]. Second, there is evidence from other angiosperms of RNA editing in ORFs and non-coding sequences, including introns, UTRs, and IGSs [37, 38]. We identified a total of 97 unique RNA editing sites in non-coding regions (Table 3), which is substantially more than the 37 reported in *Brassica oleracea* [37] and the 73 reported in *Nicotiana tabacum* [38]. However, given the much larger total amount of transcribed non-coding sequence in the 7-Mb *S. noctiflora* mitochondrial genome, the density of non-coding RNA editing sites is actually lower. One possible explanation for this pattern is that the overall complement of editing factors (pentatricopeptide repeat proteins) is smaller in *S. noctiflora* because of its reduced frequency of RNA editing in mitochondrial coding sequences [47], resulting in a lower rate of off-target editing.

## Conclusions

While our data provide clear evidence of transcription and RNA editing in many IGSs within the massive *S. noctiflora* mitochondrial genome, it remains difficult to definitively conclude whether any of these transcribed regions play an important functional role that could explain their origin or maintenance. Future work, including functional characterization of candidate non-coding RNAs and proteomic analysis of candidate ORFs may provide further insight [59–61]. In addition, studying patterns of evolutionary conservation in DNA sequence and structure remains one of our most robust tools for detecting functionally important sequences. This approach may have limited applications within *S. noctiflora* because of its low levels of intraspecific sequence diversity [9, 22]. However, it may be fruitful in other species – such as the congener *S. conica* – with similar genome architecture but higher levels of intraspecific variation [9]. Such efforts have the potential to advance our understanding of the mechanisms driving the evolution of the largest organelle genomes ever identified.

## Methods

### RNA extraction

The *Silene noctiflora* plants used for RNA extraction were full-sib F2s derived from two generations of self-crosses beginning with a single individual from the BRP maternal family that was previously used for whole mitochondrial genome sequencing [22]. Seeds were germinated on soil (Fafard 2SV Mix supplemented with vermiculite and perlite) in SC7 Cone-tainers (Stuewe and Sons) in February 2014. Plants were grown for 8 months under supplemental lighting (16-hr/8-hr light/dark cycle) with regular watering and fertilizer treatments in the Colorado State University greenhouse. During this time, the plants were cut back on multiple occasions.

Mitochondrial-enriched RNA samples were prepared by using differential centrifugation. Biological replicates were generated by separating plants into two groups. Approximately 60 g of rosette leaf tissue was sampled from each group, homogenized in a high-salt isolation buffer (containing 1.25 M NaCl, 50 mM Tris HCl (pH 8.0), 5 mM EDTA, 0.5 % polyvinylpyrrolidone, 0.2 % bovine-serum albumin, and 15 mM beta-mercaptoethanol) in a blender, and filtered through four layers of cheesecloth and one layer of Miracloth. The filtrate was centrifuged at low speed (1000 × g) for 10 min in a Beckman J2-21 centrifuge, and the resulting supernatant was centrifuged at medium speed (3000 × g) for 10 min. The supernatant from the medium speed spin was then centrifuged at high speed (10,000 × g) for 20 min to pellet mitochondria. The pellet was then gently resuspended in high-salt isolation buffer, and the above medium- and high-speed centrifugation steps were repeated to produce a final enriched mitochondrial pellet. All steps were performed at 4 °C in a refrigerated centrifuge or cold room. An intact leaf was also collected from a single individual in each group to extract total cellular RNA. To minimize loss of small RNAs, TRI Reagent (Sigma-Aldrich) was used to isolate RNA from enriched mitochondrial pellets and whole-leaf samples, followed by treatment with DNase I (Thermo Scientific) to remove contaminating DNA. The RNA was further purified by phenol:chloroform extraction and precipitated with ethanol. Prior to sequencing, RNA quality was assessed on an Agilent 2200 TapeStation, and enrichment of mitochondrial RNA was verified by quantitative reverse-transcriptase PCR (qRT-PCR).

### Illumina sequencing and read trimming

Each RNA sample was used for both mRNA-seq and small RNA-seq strand-specific library construction, which was performed at the Yale Center for Genome Analysis (YCGA). For the mRNA-seq libraries, rRNA depletion was performed with the Ribo-Zero Plant Leaf Kit (Epicentre/Illumina) to avoid biasing the representation of mitochondrial transcripts with polyA selection. For the small RNA-seq libraries, a gel cut was performed to obtain transcripts shorter than 30 nt. The resulting libraries were sequenced at YCGA on an Illumina HiSeq 2500 platform in single-read lanes with 101 and 76 bp read lengths for the mRNA and small-RNA libraries, respectively. The resulting sequencing reads were submitted to the NCBI Sequence Read Archive (SRX1153098; SRX1153129; SRX1153130; SRX1153131).

For all mRNA-seq and small RNA-seq data, read quality was assessed using FastQC version 0.10.1 (http://www.bioinformatics.babraham.ac.uk/projects/fastqc/). Adapter and low-quality sequences were trimmed using Trimmomatic version 0.32 [62] with the following modified parameters: −phred33 ILLUMINACLIP: TruSeq3-SE.fa:2:30:10 LEADING:3 TRAILING:3 SLIDINGWINDOW:4:15 MINLEN:50.

### mRNA-seq data analysis

We employed Bowtie2 v2.2.4 [63] to map the filtered reads from each library to the mitochondrial genome of *S. noctiflora* BRP (GenBank KP053825-KP053887) with default parameters. Samtools v1.2 (http://www.htslib.org/) [64] was used to calculate read depth at each position in the genome from the resulting SAM files and convert them into separate forward-strand and reverse-strand BAM files. To estimate coverage of each annotated mitochondrial gene in terms of reads per kilobase per million mapped reads (RPKM), the filtered read files and the reference mitochondrial genome were imported into CLC Genomics Workbench v7.5.1 for coverage analysis with default parameters.

Geneious v7.1.5 (Biomatters Ltd.) was used to identify all ORFs with a minimum length of 201 bp, and custom Perl scripts were used to extract mean read depth for each ORF from the BAM files. We selected the 5 % subset of ORFs with the highest mean read depth for further analysis as candidates for expressed functional elements and used these sequences as queries for NCBI-BLASTP and BLASTN v2.2.28^+^ searches against the NCBI nr and nt databases, which were performed with an E-value threshold of 0.001. The BLASTN (MEGABLAST) search was run with a non-default word size of 11 and the dust option disabled. Candidate ORFs were searched for potential transmembrane domains using TMHMM 2.0c [65]

### Small RNA data analysis

Small RNAs shorter than 17 nt or longer than 25 nt were discarded from each of the four libraries. Bowtie2 was employed to map the small RNA reads to the reference mitochondrial genome, and Samtools was used to filter for perfectly matched reads and generate strand-specific BAM files for each library. To identify candidate functional small RNAs, we restricted our analysis to reads that 1) were not found on the coding strand of annotated protein, tRNA, or rRNA genes, 2) mapped to the exact same location with the same length and no more than one overlapping read from neighboring positions, 3) had a minimum depth of 50×. The resulting candidate small RNAs were used to search for similar sequences using the BLASTN implementation in miRBase release 21 (http://www.mirbase.org/) [66].

### RNA editing site detection

To find potential RNA editing sites, we identified mismatches between reads from the mitochondrial-enriched mRNA-seq libraries and the reference genome. BAM files generated from Bowtie2 mapping were used as inputs for the variant detection function in CLC Genomics Workbench to call mismatches with a minimum read depth of 100× and a minimum variant frequency of 20 %. Because of the challenge associated with mapping reads spanning intron/exon boundaries, we excluded putative intronic editing sites within 20 bp of such boundaries. Read mapping data were all manually inspected using IGV [67] to verify candidate sites.

### Availability of supporting data

All Illumina RNA-seq reads were deposited to the NCBI Short Read Archive (SRX1153098; SRX1153129; SRX1153130; SRX1153131).

## Additional file

Additional file 1: Figures S1-S9 and Tables S1-S6. (PDF 5853 kb)

## Abbreviations

UTRs: Untranslated regions
IGT: Intracellular gene transfer
HGT: Horizontal gene transfer
IGS: Intergenic sequence
ORFs: Open reading frames
CMS: Cytoplasmic∪ male sterility
RNA-seq: High-throughput cDNA sequencing
mRNA-seq: Conventional RNA-seq
C-to-U: Cytidine-to-uridine
qRT-PCR: Quantitative reverse-transcriptase PCR
RPKM: Reads per kilobase per million mapped reads
rRNA: Ribosomal RNA
tRNA: Trnasfer RNA
