# Supplementary material for: High transcript abundance, RNA editing, and small RNAs in intergenic regions within the massive mitochondrial genome of the angiosperm Silene noctiflora
Source: BMC Genomics. 2015 Nov 14;16:938. doi: 10.1186/s12864-015-2155-3 (PMC4647634; doi:10.1186/s12864-015-2155-3)
Supplement: Additional file 1: Figures S1-S9 and Tables S1-S6. — (PDF 5853 kb) [file 12864_2015_2155_MOESM1_ESM.pdf]

Figure S1

Correlation between RNA-seq read depths for pairs of biological replicates. Coverage estimates (in terms of average read depth) are based on a sliding window with a window size of 200 bp and a step size of 100 bp. (A) mitochondrial-enriched mRNA-seq libraries; (B) total-cellular mRNA-seq libraries; (C) mitochondrial-enriched small RNA-seq libraries; (D) total-cellular small RNA-seq libraries. The red points represent annotated gene regions, including introns and 2 kb of 5′ and 3′ flanking sequences. The *r*-values represent the Pearson's correlation coefficient as calculated in R. 1:1 lines are shown in blue.

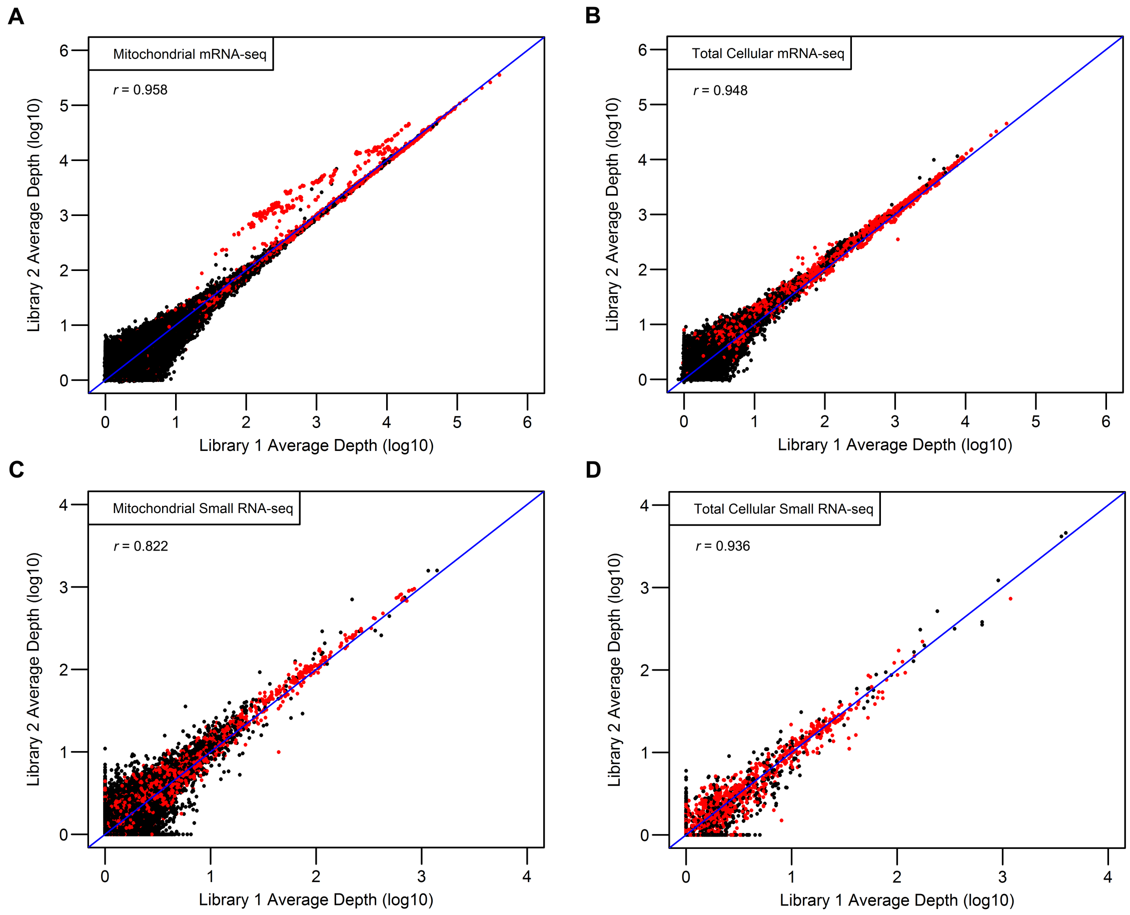

Figure S2

Illustration of strand specificity in mRNA-seq libraries. Coverage estimates (in terms of average read depth) are based on a sliding window with a window size of 100 bp and a step size 50 bp for the average of two replicates of mitochondrial-enriched libraries. Coverage values for forward and reverse strands were displayed above and below the x-axis, respectively. The pink shading indicates the boundaries of protein-coding genes/exons, and the gray arrows indicate the orientation of the coding strand.

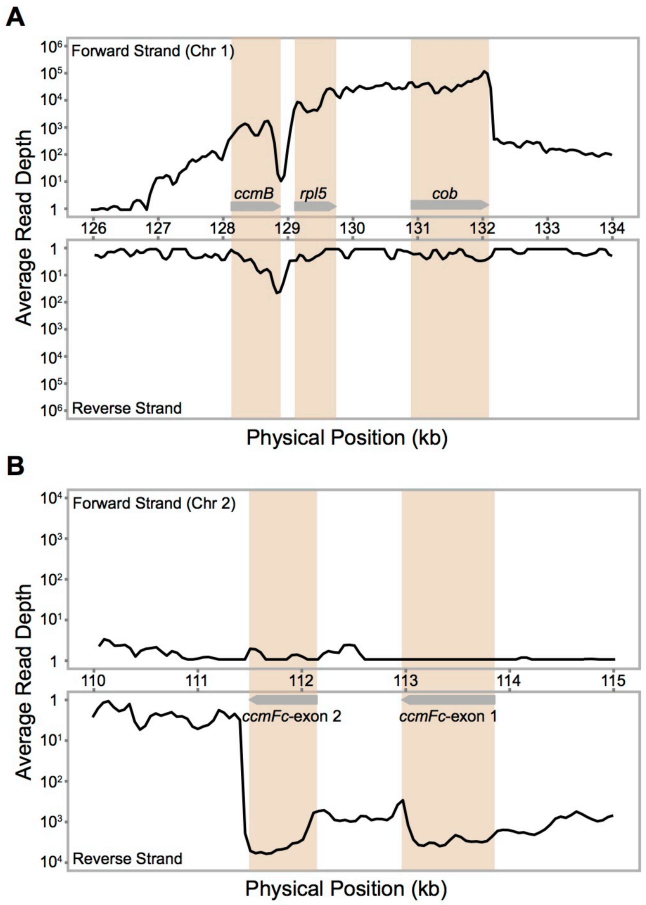

Figure S3

The decline in mRNA-seq coverage prior to the first in-frame stop codon in *nad6* and *ccmC*. Coverage estimates (in terms of average read depth) are based on a sliding window with a window size of 20 bp and a step size of 10 bp for the average of two replicates of mitochondrial-enriched libraries The pink shading indicates the boundaries of the protein-coding genes, and the gray arrows indicate the orientation of the coding strand.

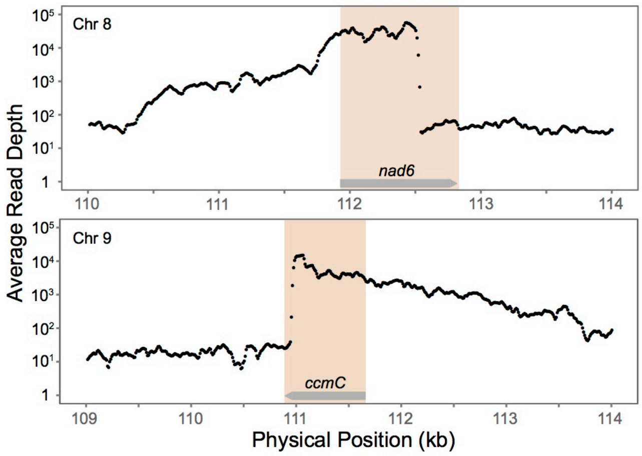

Figure S4

The full alignment of the intact *rpl5* copy on chromosome 1 and the *rpl5* pseudogene on chromosome 24.

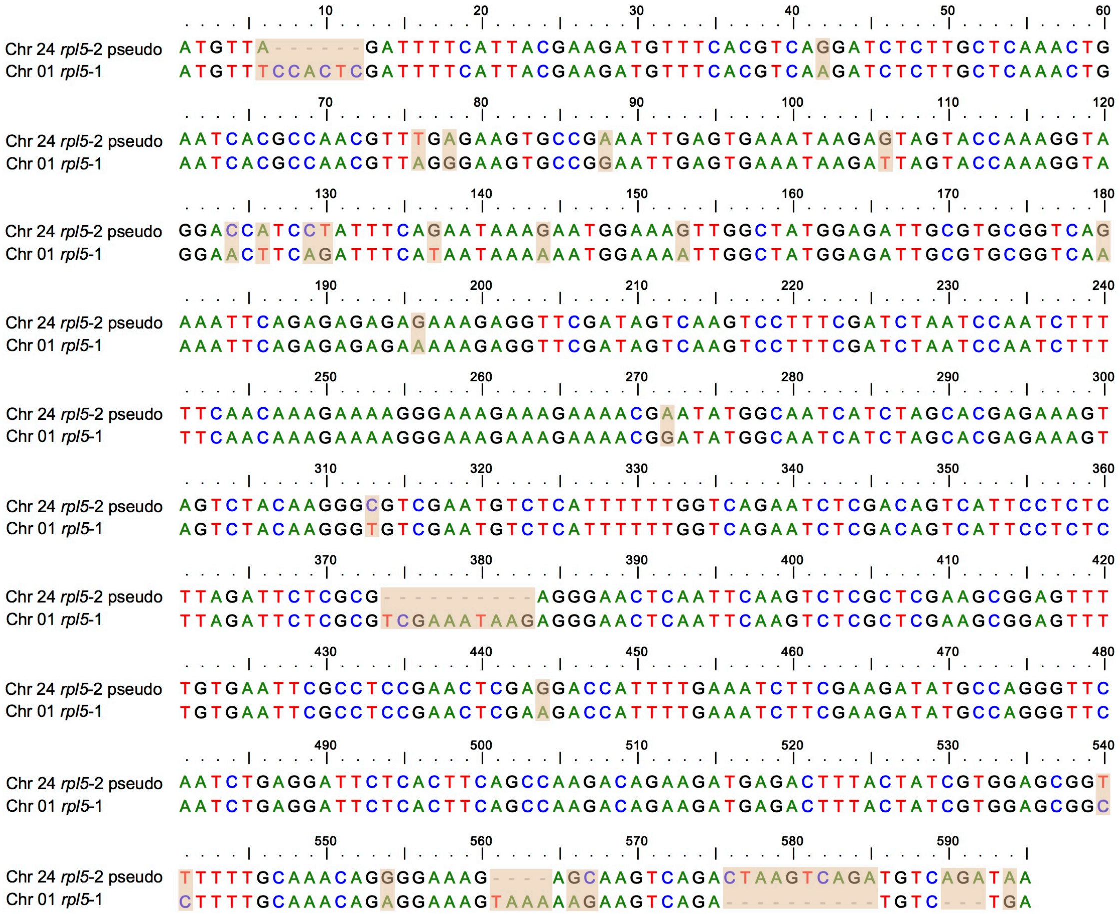

Figure S5

Examples of pseudogenes containing internal regions that are perfectly identical to the intact copy of the gene and are therefore subject to cross-mapping of reads that originate from the intact gene copy. Coverage estimates (in terms of average read depth) are based on a sliding window with a window size of 100 bp and a step size 50 bp for the average of two replicates of mitochondrial-enriched libraries. The pink shading indicates the boundaries of pseudogenes/exons, and the gray arrows indicate the orientation of the coding strand. Internal regions corresponding to coverage spikes in the (A) *mttB* and (B) *nad2*-exon4 pseudogenes are identical to the corresponding functional genes.

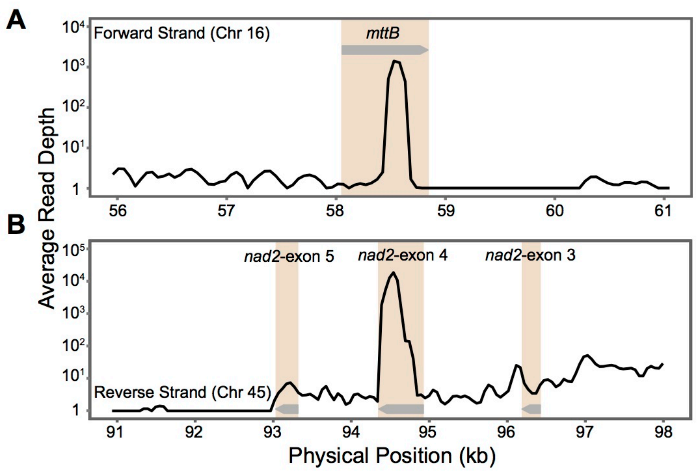

Figure S6

The frequency distribution of average mRNA-seq read depth. Read depths are calculated based on 500-bp windows across the entire mitochondrial genome for the average of two replicates of mitochondrial-enriched libraries. The 5% tail of the distribution corresponds to windows with coverage >70×. Windows with >100× are found in the 4% tail of the distribution.

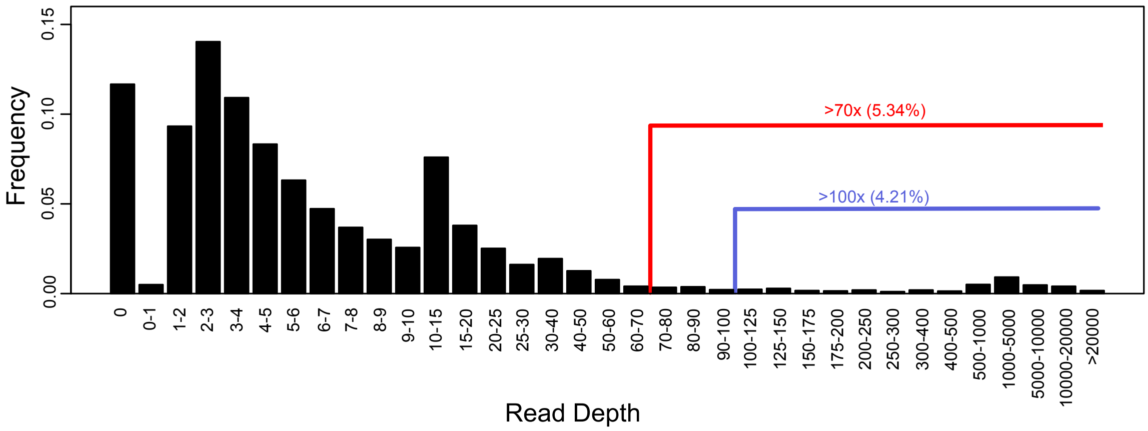

Figure S7

Mitochondrial enrichment for mRNA-seq relative to total-cellular libraries across the 63 chromosomes of the *Silene noctiflora* mitochondrial genome. This plot is a duplicate of Figure 1 except that points are color-coded based on their degree of overrepresentation in the mitochondrial-enriched libraries. Red and green points indicate overrepresentation in the mitochondrial-enriched and total-cellular libraries, respectively. Color scaling is based on the ratio of mitochondrial-enriched to total-cellular read depths (in terms of counts per million) expressed on a $\log_{10}$ scale. Expression values were set to a minimum of 1 for these calculations.

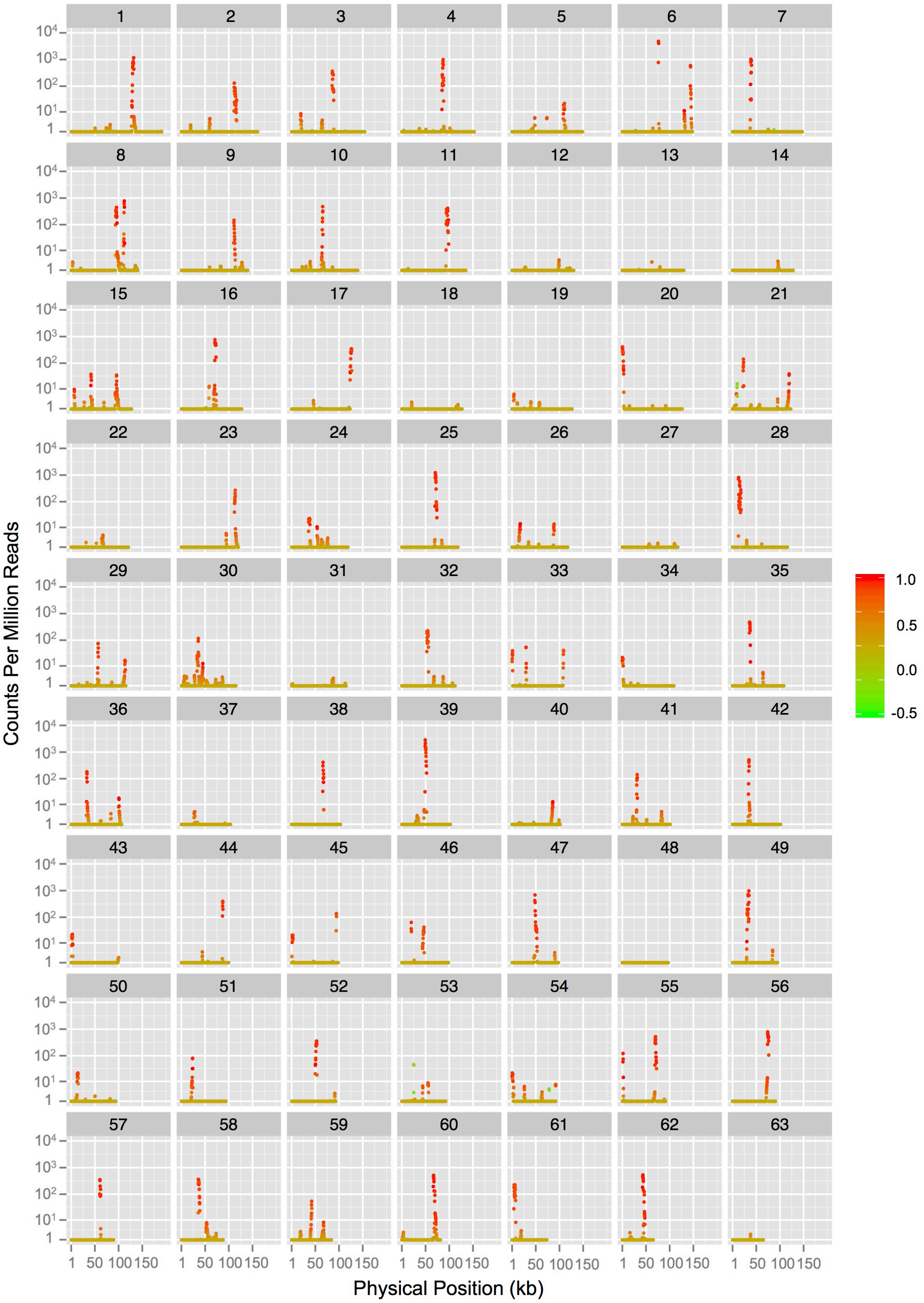

Counts Per Million Reads
Physical Position (kb)
1 50 100 150
10^4 10^3 10^2 10^1 1
1.0 0.5 0.0 -0.5
1 2 3 4 5 6 7 8 9 10 11 12 13 14 15 16 17 18 19 20 21 22 23 24 25 26 27 28 29 30 31 32 33 34 35 36 37 38 39 40 41 42 43 44 45 46 47 48 49 50 51 52 53 54 55 56 57 58 59 60 61 62 63

Figure S8

Mitochondrial enrichment for small RNA-seq relative to total-cellular libraries across the 63 chromosomes of the *Silene noctiflora* mitochondrial genome. This plot is a duplicate of Figure 3 except that points are color-coded based on their degree of overrepresentation in the mitochondrial-enriched libraries. Red and green points indicate overrepresentation in the mitochondrial-enriched and total-cellular libraries, respectively. Color scaling is based on the ratio of mitochondrial-enriched to total-cellular read depths (in terms of counts per million) expressed on a $\log_{10}$ scale. Expression values were set to a minimum of 1 for these calculations.

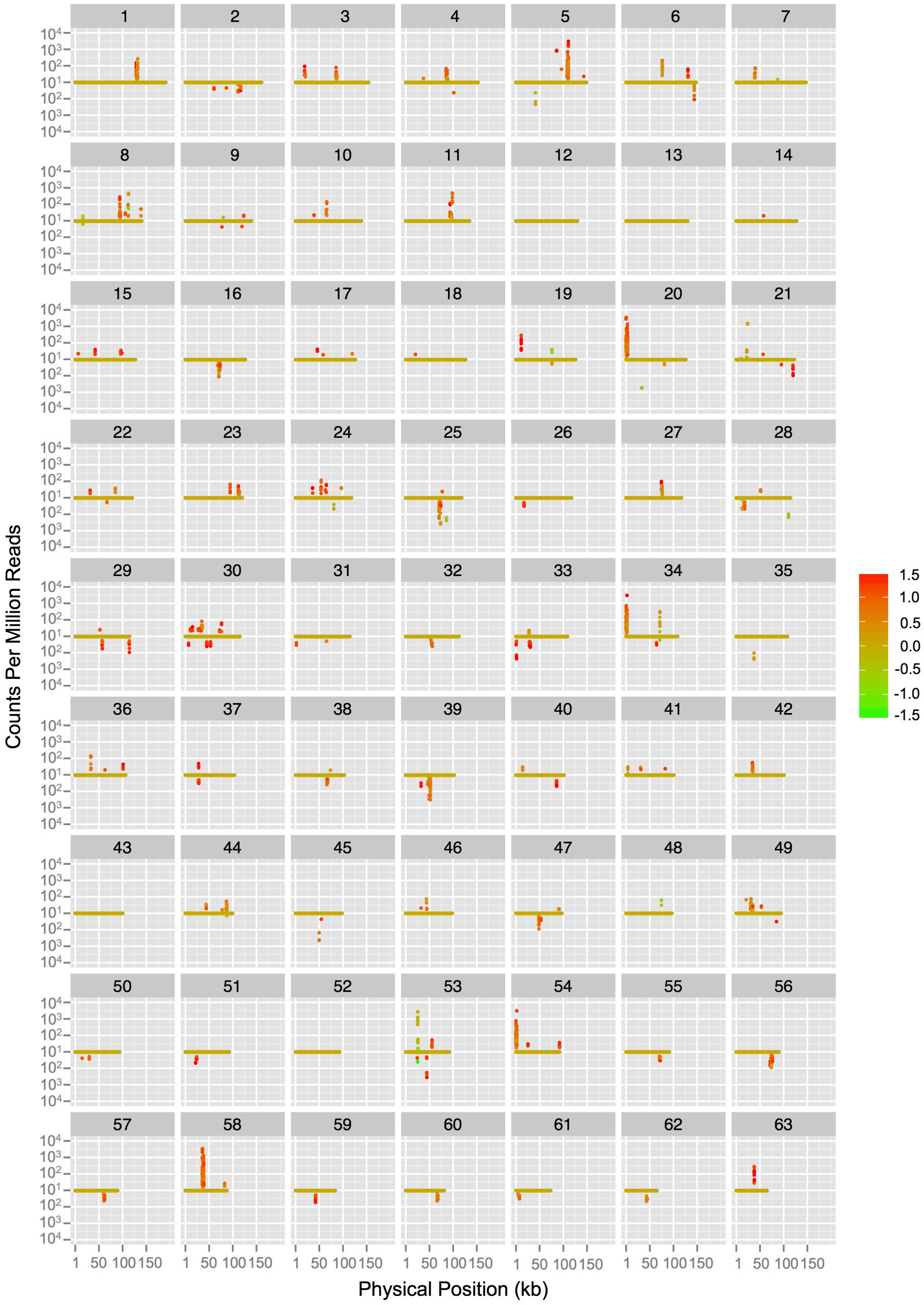
1 2 3 4 5 6 7 8 9 10 11 12 13 14 15 16 17 18 19 20 21 22 23 24 25 26 27 28 29 30 31 32 33 34 35 36 37 38 39 40 41 42 43 44 45 46 47 48 49 50 51 52 53 54 55 56 57 58 59 60 61 62 63
Counts Per Million Reads
Physical Position (kb)
1 50 100 150
1.5
1.0
0.5
0.0
-0.5
-1.0
-1.5

Figure S9

Correlation between RNA-seq coverage from mRNA and small-RNA libraries. Coverage estimates (in terms of average read depth) are based on a sliding window with a window size of 200 bp and a step size of 100 bp. Forward and reverse strands within each window were analyzed separately. The red points represent annotated gene regions, including introns and 2 kb of 5′ and 3′ flanking sequences. The *r*-value is the Pearson’s correlation coefficient as calculated in R. The 1:1 line is shown in blue.

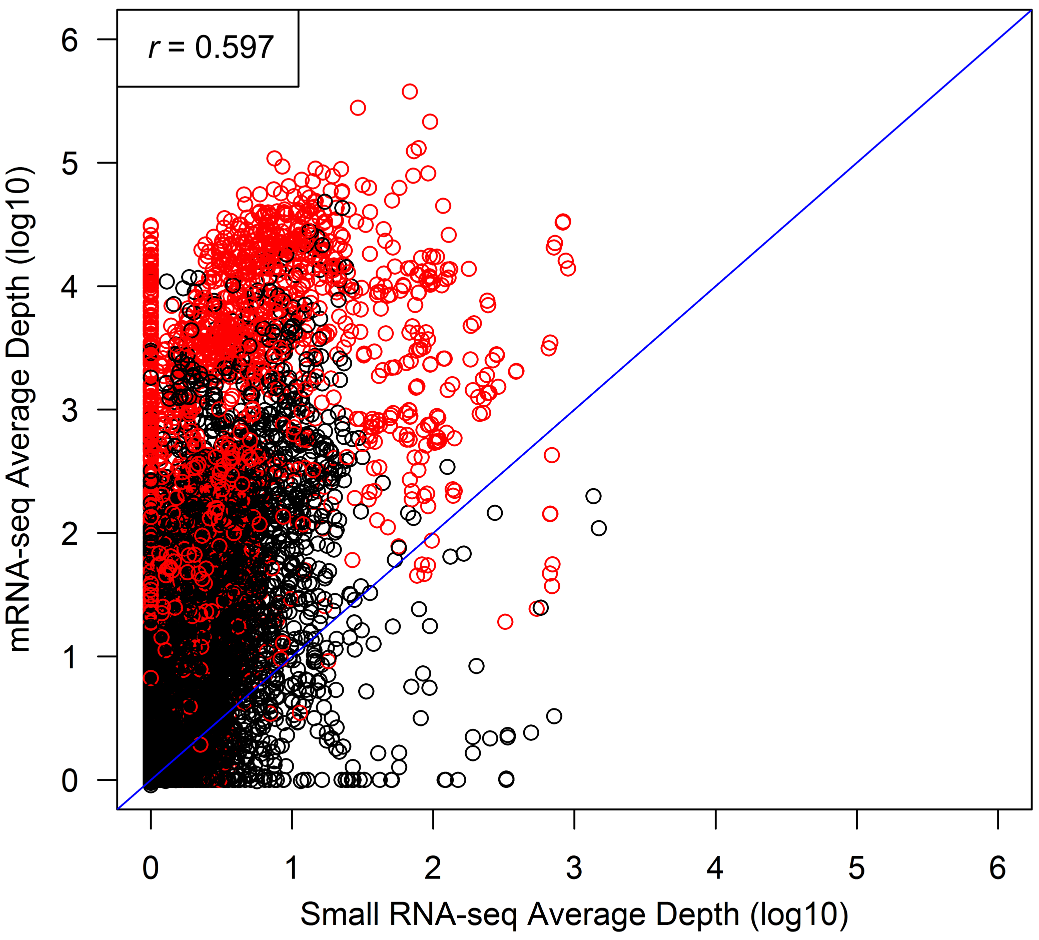

Table S1. Summary of average read depth based on raw read counts for each gene

Chrom	Gene	Start	End	Strand	Length (bp)	Mitochondrial Coverage			Total Cellular Coverage			
						Lib 1	Lib 2	Mean	Lib 1	Lib 2	Mean	Ratio
1	*ccmB-a*	128198	128818	+	621	1016	980	998	103	124	113	8.80
1	*rpl5-1*	129131	129712	+	582	11289	10419	10854	1195	1416	1306	8.31
1	*cob*	130875	132044	+	1170	42423	40005	41214	4449	4800	4624	8.91
2	*ccmFc-exon2*	111512	112061	-	550	4895	4971	4933	584	615	599	8.23
2	*ccmFc-exon1*	112986	113752	-	767	3325	3407	3366	456	467	461	7.30
3	*nad1-exon2*	86375	86456	+	82	9216	7772	8494	1095	1197	1146	7.41
3	*nad1-exon3*	87835	88026	+	192	25995	23259	24627	2998	3204	3101	7.94
4	*nad5-exon1*	85304	85533	+	230	14953	13304	14129	1513	1775	1644	8.60
4	*nad5-exon2*	86399	87614	+	1216	33334	31517	32425	3588	4084	3836	8.45
5	*trnfM(CAT)*	108843	108917	+	75	273	252	263	12	20	16	16.37
5	*rrn18-a*	109091	110881	+	1791	244	1166	705	80	76	78	9.05
5	*rrn5-a*	111077	111194	+	118	306	327	316	65	70	67	4.69
6	*atp9*	76509	76733	+	225	379607	339348	359477	36120	42849	39484	9.10
6	*nad1-exon5*	144016	144274	-	259	36648	34344	35496	4006	4575	4290	8.27
7	*nad5-exon4*	38470	38864	+	395	54706	53330	54018	5670	6406	6038	8.95
7	*nad5-exon5*	39889	40044	+	156	79211	73720	76466	7588	8909	8249	9.27
8	*nad2-exon1*	94355	94507	+	153	17147	14857	16002	1860	2108	1984	8.07
8	*nad2-exon2*	95484	95875	+	392	22734	20649	21692	2543	2891	2717	7.98
8	*nad6*	111927	112811	+	885	21747	20715	21231	2132	2086	2109	10.07
9	*ccmC*	110920	111654	-	735	5523	5478	5501	724	766	745	7.38
10	*nad9*	65434	66012	+	579	19091	18461	18776	2119	2410	2264	8.29
11	*nad4-exon1*	94197	94657	+	461	13852	13346	13599	1577	1793	1685	8.07
11	*nad4-exon2*	96212	97149	+	938	15458	14569	15014	1698	1935	1817	8.26
11	*nad4-exon3*	98419	98507	+	89	28525	25300	26912	2644	3137	2891	9.31
16	*cox1-a*	70929	72512	-	1584	24656	22876	23766	2315	2842	2578	9.22
17	*rrn26-a*	123577	126684	-	3108	5406	12276	8841	1137	1077	1107	7.99
20	*rrn26-b*	226	3333	+	3108	5471	12635	9053	1148	1113	1131	8.01
21	*nad5-exon3*	22965	22986	+	22	3129	3106	3117	429	533	481	6.48
23	*mttB-1*	113128	113916	+	789	9031	8496	8764	1260	1595	1428	6.14
25	*cox3*	70464	71261	-	798	49339	44986	47162	4603	5704	5153	9.15
25	*atp8*	71652	72155	-	504	28094	25678	26886	2906	3573	3239	8.30
28	*nad7-exon4*	12793	13054	-	262	49837	48556	49196	5213	5844	5528	8.90
28	*nad7-exon3*	14898	15608	-	711	23235	21765	22500	2331	2660	2496	9.02
28	*nad7-exon2*	16443	16511	-	69	1976	1755	1866	213	293	253	7.38
28	*nad7-exon1*	17338	17480	-	143	12140	12220	12180	1377	1502	1440	8.46
32	*matR-a*	52975	54972	-	1998	6493	6258	6375	897	1017	957	6.66
32	*nad1_exon4*	56141	56199	-	59	4442	4183	4312	573	999	786	5.49
34	*rrn18-b*	229	2019	+	1791	241	1165	703	78	75	76	9.21
34	*rrn5-b*	2215	2332	+	118	314	313	313	61	74	68	4.62
35	*nad1-exon1*	36866	37251	-	386	12093	11220	11657	1186	1395	1290	9.03
36	*nad3-a*	33294	33650	+	357	7030	6752	6891	743	740	742	9.29
39	*atp6*	49368	50297	-	930	75277	74101	74689	7375	8979	8177	9.13
39	*atp1*	50507	52027	-	1521	29066	31061	30063	3456	4093	3774	7.97
42	*rps13*	34852	35202	+	351	22480	21292	21886	2383	2622	2503	8.75
43	*rrn5-c*	968	1085	-	118	316	312	314	65	71	68	4.59
43	*rrn18-c*	1281	3071	-	1791	242	1154	698	81	76	78	8.90
43	*trnfM(CAT)*	3245	3319	-	75	304	243	273	15	27	21	13.03
44	*trnI-CAT*	55795	55868	+	74	56	39	48	--	--	--	--
45	*rrn5-d*	874	991	-	118	333	314	324	57	60	59	5.52
45	*rrn18-d*	1187	2977	-	1791	241	1152	696	77	75	76	9.16
46	*ccmFn*	45188	46864	+	1677	1273	1296	1285	188	230	209	6.15
49	*trnY-GTA*	30558	30639	+	82	213	220	216	11	19	15	14.25
49	*nad2-exon3*	31145	31305	+	161	19554	17501	18527	2124	2451	2287	8.10
49	*nad2-exon4*	32891	33463	+	573	29865	29156	29510	3194	3705	3450	8.55

49	*nad2-exon5*	34662	34849	+	188	92370	92099	92235	9733	10912	10323	8.94
50	*rrn5-e*	12422	12539	-	118	308	302	305	75	57	66	4.62
50	*rrn18-e*	12735	14525	-	1791	240	1167	703	78	77	77	9.11
52	*rrn26-c*	50094	53201	-	3108	5364	12381	8872	1128	1070	1099	8.07
54	*rrn18-f*	228	2018	+	1791	242	1162	702	80	77	78	8.96
54	*rrn5-f*	2214	2331	+	118	358	357	357	78	69	73	4.87
54	*trnI-CAT*	80167	80240	+	74	22	16	19	--	--	--	--
55	*nad3-b*	1634	1990	-	357	6356	6264	6310	691	671	681	9.27
55	*cox2*	69579	70355	-	777	18519	17250	17884	1950	2399	2174	8.23
56	*cox1-b*	74308	75891		1584	24818	22829	23823	2306	2836	2571	9.27
58	*rrn26-d*	35817	38924	+	3108	5484	12531	9008	1159	1110	1134	7.94
59	*trnI-CAT*	67221	67294	+	74	245	217	231	--	--	--	--
60	*atp4-a*	67171	67875	-	705	15786	15196	15491	1486	1815	1651	9.38
60	*nad4L-a*	67923	68225	-	303	12485	11350	11917	1096	1341	1219	9.78
60	*ccmB-b*	70309	70929	-	621	747	703	725	76	86	81	8.94
61	*matR-b*	4240	6237	-	1998	6525	6223	6374	912	1003	957	6.66
61	*nad1-exon4*	6892	6950	-	59	2402	2194	2298	345	448	396	5.80
61	*nad5-exon3*	39569	39590	-	22	1	1	1	1	1	1	--
62	*atp4-b*	43681	44268	-	588	16060	15578	15819	1518	1807	1662	9.52
62	*nad4L-b*	44443	44735	-	293	12298	11409	11853	1114	1375	1245	9.52
62	*ccmB-c*	46819	47439	-	621	762	692	727	69	86	78	9.37

Table S2. Summary of coverage based on RPKM for each gene

						Mitochondrial RPKM		Total Cellular RPKM	
**Chrom**	**Gene**	**Start**	**End**	**Strand**	**Length (bp)**	**Lib 1**	**Lib 2**	**Lib 1**	**Lib 2**
1	*ccmB-a*	128198	128818	+	621	1813	1783	1266	1314
1	*cob*	130875	132044	+	1170	25680	25208	20076	17577
1	*rpl5-1*	129131	129712	+	582	13045	12692	10258	9763
2	*ccmFc*	111512	113752	-	2241	1852	2011	1766	1580
3	*nad1_exon2-3*	86375	88026	+	1652	5181	4949	4824	4406
4	*nad5_exon1-2*	85304	87614	+	2311	15193	15122	12151	11354
6	*atp9*	76509	76733	+	225	243232	230507	176175	170519
6	*nad1_exon5*	144016	144274	-	259	20087	19608	16931	15194
7	*nad5_exon4-5*	38470	40044	+	1575	16403	16679	12693	11516
8	*nad2_exon1-2*	94355	95875	+	1521	11265	10770	9425	9530
8	*nad6*	111927	112811	+	885	15394	15483	11516	9043
9	*ccmC*	110920	111654	-	735	4004	4166	3933	3395
10	*nad9*	65434	66012	+	579	12017	12345	10037	9487
11	*nad4*	94197	98507	+	4311	5369	5354	4441	4362
16	*cox1*	70929	72512	-	1584	34518	33500	23713	23823
21	*nad5_exon3*	22965	22986	+	22	0	0	0	0
23	*mttB-1*	113134	113916	+	783	6062	6017	6325	6635
25	*atp8*	71652	72155	-	504	17520	17038	14115	13698
25	*cox3*	70464	71261	-	798	35428	34078	24954	24963
28	*nad7*	12793	17480	-	4688	6130	6175	4873	4500
32	*matR-a*	52975	54972	-	1998	8846	8904	9157	8382
32	*nad1_exon4*	56141	56199	-	59	0	0	0	0
35	*nad1_exon1*	36866	37251	-	386	7958	7496	5878	5623
36	*nad3-a*	33294	33650	+	357	6908	6941	5863	4446
39	*atp1*	50507	52027	-	1521	20089	22890	17674	17172
39	*atp6*	49368	50297	-	930	57870	60447	42940	41979
42	*rps13*	34852	35202	+	351	10921	10747	8927	8004
46	*ccmFn*	45188	46864	+	1677	907	992	958	970
49	*nad2_exon3-5*	31145	34849	+	3705	9539	9557	7770	7518
55	*cox2*	69579	70355	-	777	17946	17549	14080	14144
60	*atp4-a*	67171	67758	-	588	21682	22170	15217	15058
60	*nad4L-a*	67923	68225	-	303	14629	14154	9709	9343
61	*nad1_exon4*	6892	6950	-	59	2	7	29	0
61	*nad5_exon3*	39569	39590	+	22	0	0	0	0

Table S3. Summary of ORFs with a minimum length of 201 bp and a minimum average read depth of 100 in the mitochondrial-enriched mRNA-seq libraries.

				Mean Read Depth			
Chrom	Start	End	Strand	Library 1	Library 2	Length (bp)	BlastP
3	20517	21017	+	150	143	501	NADH dehydrogenase subunit 7
5	48135	48359	+	412	403	225	-
5	73579	73800	-	448	402	222	-
6	130396	130677	+	183	169	282	-
6	131782	132084	+	154	138	303	-
8	99294	99596	+	203	170	303	-
15	6443	6715	+	314	290	273	-
15	27836	28039	-	154	173	204	-
15	41526	41759	+	399	377	234	-
15	42451	42810	+	1278	1140	360	NADH dehydrogenase subunit 2
15	93838	94122	+	237	230	285	-
15	94663	94896	+	213	183	234	-
15	95017	95226	+	174	152	210	-
15	96791	97009	+	265	263	219	-
15	98089	98313	+	130	105	225	-
16	58291	58818	+	346	303	528	mttB
19	11206	11457	+	145	166	252	-
21	8198	8425	+	422	398	228	-
21	9977	10207	+	803*	699*	231	photosystem I P700 apoprotein A1
21	119670	119927	-	1710*	1516*	258	-
22	66400	66672	-	109	112	273	-
24	36185	36571	+	898	855	387	ribosomal protein L5
24	54187	54402	+	280	281	216	-
24	54475	54879	+	163	139	405	-
26	88247	88462	+	453	513	216	-
29	55864	56082	-	210*	171*	219	-
29	112522	112725	-	108	103	204	-
29	113026	113256	-	274	245	231	-
29	113833	114057	-	678	627	225	-
29	114123	114335	-	933	862	213	-
30	34569	34913	+	695	584	345	-
30	35484	36026	+	4516	4246	543	NADH dehydrogenase subunit 2
30	43555	43926	-	128	121	372	-
30	44377	44598	-	234	216	222	-
30	45214	45468	-	607	594	255	-
32	87167	87445	+	124	128	279	-
32	107059	107310	-	116	112	252	-
33	358	708	-	835	750	351	-
33	29653	29907	-	1170	986	255	-
33	30251	30451	-	231	195	201	-
36	101925	102140	+	171	159	216	-
37	26965	27177	-	136	129	213	-
38	66657	67385	-	4487	4419	729	ATPase subunit 6
40	84633	84929	-	168	142	297	-
40	85766	85966	-	526	459	201	-

41	81772	82023	-	124	124	252	-
44	86758	87297	+	11959*	11307*	540	cytochrome c oxidase subunit 2
44	87703	87909	+	140*	130*	207	-
45	94347	94790	-	6513	5992	444	NADH dehydrogenase subunit 2
47	47498	47785	-	133	115	288	-
47	48353	49729	-	14286	12960	1377	ribosomal protein S3
47	50250	50528	-	1768	1547	279	-
47	50593	50820	-	1233	1072	228	-
47	52328	52570	-	779	643	243	-
49	84844	85062	-	253	230	219	-
53	55930	56151	+	486	428	222	NADH dehydrogenase subunit 7
54	63317	63526	-	190	175	210	-
57	61121	61849	-	4456	4338	729	ATPase subunit 6
58	53923	54138	+	149	142	216	-
59	41368	41589	-	490	427	222	NADH dehydrogenase subunit 7
59	65656	65946	-	108	112	291	-
60	3277	3486	-	119	126	210	-
63	38053	38304	+	151	165	252	-

*Coverage values may be inflated because the ORF overlaps with a region that is duplicated or highly similar to another gene.

Table S4. Summary of all identified C-to-U RNA editing sites. Sites labeled as intron/IGS were found adjacent to an exon with a *trans*-spliced intron with unknown boundaries, so it is not clear whether they are in the intron or IGS.

Chrom	Position	Strand	Gene/Location	Coverage	Edited (%)	Mean Quality
1	128225	+	ccmB-a	706	99.4	37.5
1	128267	+	ccmB-a	564	96.8	38.2
1	128268	+	ccmB-a	564	96.8	38.2
1	128351	+	ccmB-a	165	97.6	34.5
1	128357	+	ccmB-a	212	88.7	33.6
1	128361	+	ccmB-a	262	95.8	33.5
1	128501	+	ccmB-a	523	99.4	38.0
1	128664	+	ccmB-a	898	97.2	39.0
1	128691	+	ccmB-a	550	97.8	35.7
1	128700	+	ccmB-a	269	82.9	36.8
1	129165	+	rpl5	9124	92.0	37.8
1	129194	+	rpl5	9213	92.8	37.7
1	129474	+	rpl5	3879	94.1	37.5
1	129660	+	rpl5	27632	90.9	37.1
1	131199	+	cob	31293	98.7	38.2
1	131232	+	cob	19165	98.9	35.7
1	131442	+	cob	26100	99.5	39.1
1	131682	+	cob	8374	98.9	34.9
1	131782	+	cob	35816	98.3	37.6
1	132034	+	cob	126110	99.8	39.2
2	111595	-	ccmFc	6047	99.7	38.1
2	111669	-	ccmFc	8243	98.1	37.0
2	113335	-	ccmFc	3976	99.6	36.3
2	113598	-	ccmFc	3832	91.7	37.4
2	113602	-	ccmFc	3745	99.2	37.0
2	113630	-	ccmFc	2659	87.9	38.2
2	113703	-	ccmFc	3529	98.1	35.4
2	113819	-	5UTR-ccmFc	2148	92.5	35.9
2	113910	-	5UTR-ccmFc	1762	20.4	37.3
2	114327	-	5UTR-ccmFc	1495	83.7	37.8
2	115766	-	IGS	502	24.3	36.5
2	115832	-	IGS	527	95.5	36.9
2	115884	-	IGS	230	86.1	36.8
3	19664	+	IGS	336	58.3	39.0
3	19842	+	IGS	428	68.5	38.3
3	87856	+	nad1-3	10633	95.6	36.2
3	87866	+	nad1-3	15401	89.8	36.2
3	87902	+	nad1-3	30676	94.5	36.5

3	88465	+	intron/IGS-nad1-3	2914	88.6	36.6
4	85458	+	nad5-1	10696	97.1	36.6
4	86410	+	nad5-2	19807	98.9	37.3
4	86542	+	nad5-2	24318	99.4	38.3
4	86566	+	nad5-2	33424	99.5	36.6
4	86716	+	nad5-2	21415	97.5	36.8
4	86721	+	nad5-2	23496	96.9	36.6
4	86776	+	nad5-2	26054	99.5	38.2
4	86893	+	nad5-2	30369	98.8	33.8
4	87003	+	nad5-2	37968	99.6	35.6
4	87478	+	nad5-2	65369	99.6	36.2
4	87896	+	intron/IGS-nad5-2	7864	66.7	36.8
5	73794	-	IGS	191	78.0	35.5
6	76528	+	atp9	226140	99.0	35.9
6	144066	-	nad1-5	53836	99.2	36.7
6	144096	-	nad1-5	30260	99.2	37.1
6	144251	-	nad1-5	10431	98.0	34.9
6	144786	-	intron/IGS-nad1-5	2017	90.2	35.5
7	38551	+	nad5-4	54499	97.7	36.8
7	38581	+	nad5-4	92780	99.4	36.2
7	38611	+	nad5-4	71512	99.7	37.7
7	39920	+	nad5-5	41655	98.8	37.0
7	39983	+	nad5-5	69836	99.4	37.2
8	94276	+	5UTR-nad2	18527	34.0	37.0
8	94380	+	nad2-1	29476	93.7	37.9
8	95686	+	nad2-2	8556	98.1	37.5
8	95691	+	nad2-2	8508	98.2	37.1
8	95724	+	nad2-2	9708	96.8	35.6
8	95758	+	nad2-2	22091	98.4	36.3
8	95827	+	nad2-2	23403	97.8	39.0
8	111845	+	5UTR-nad6	20630	94.8	36.2
8	112014	+	nad6	41731	90.9	36.8
8	112015	+	nad6	41731	90.9	36.8
8	112021	+	nad6	40101	85.6	36.4
8	112072	+	nad6	27070	99.6	38.5
8	112117	+	nad6	12981	99.3	37.0
8	112495	+	nad6	38230	99.8	38.6
9	111015	-	ccmC	14144	99.8	39.2
9	111038	-	ccmC	14765	99.4	38.5
9	111074	-	ccmC	8482	97.7	36.5
9	111112	-	ccmC	7311	58.9	39.0
9	111189	-	ccmC	4161	97.4	37.1
9	111214	-	ccmC	3255	28.6	38.0

9	111215	-	ccmC	3255	70.1	37.6
9	111221	-	ccmC	3284	98.3	37.7
9	111225	-	ccmC	3896	98.6	37.5
9	111230	-	ccmC	4074	98.5	37.6
9	111237	-	ccmC	3815	98.3	38.3
9	111242	-	ccmC	3725	99.3	38.5
9	111293	-	ccmC	5003	99.0	37.3
9	111357	-	ccmC	3271	98.1	36.1
9	111504	-	ccmC	3977	98.4	37.3
9	111585	-	ccmC	4296	91.3	38.0
9	111715	-	5UTR-ccmC	2582	40.0	38.0
9	111892	-	5UTR-ccmC	2419	22.8	38.4
9	112186	-	5UTR-ccmC	1751	80.4	36.0
9	112543	-	5UTR-ccmC	838	41.9	37.2
10	65340	+	5UTR-nad9	5769	33.0	37.2
10	65731	+	nad9	21147	99.0	35.6
11	94270	+	nad4-1	11376	96.8	38.3
11	94303	+	nad4-1	16521	95.9	37.2
11	94354	+	nad4-1	17231	96.4	37.6
11	94393	+	nad4-1	15488	98.6	38.1
11	94564	+	nad4-1	11174	98.1	38.2
11	94629	+	nad4-1	6029	87.2	37.8
11	94633	+	nad4-1	5933	97.4	36.6
11	96760	+	nad4-2	16081	98.3	36.6
11	97105	+	nad4-2	5321	98.7	39.1
11	97573	+	intron-nad4	2777	25.7	38.4
11	97708	+	intron-nad4	1918	92.5	37.8
11	98436	+	nad4-3	29316	99.0	37.1
11	98452	+	nad4-3	29492	99.2	39.2
15	42079	+	nad2-pseudo	5715	90.9	34.3
15	42671	+	nad2-pseudo	3822	97.1	38.6
15	42676	+	nad2-pseudo	3816	97.0	39.0
15	42709	+	nad2-pseudo	919	89.9	36.7
16	58517	+	mttb-pseudo	1651	96.9	39.2
16	58525	+	mttb-pseudo	1648	94.8	39.3
19	4132	+	IGS	319	33.2	36.5
19	4323	+	IGS	617	31.3	38.6
21	22674	+	intron/IGS-nad5-3	2032	43.4	37.2
21	22785	+	intron/IGS-nad5-3	1654	81.5	37.1
23	112715	+	5UTR-mttB	3515	47.6	36.7
23	112770	+	5UTR-mttB	4991	25.1	36.9
23	113221	+	mttB	4634	92.4	37.8
23	113257	+	mttB	6540	91.5	38.0

23	113288	+	mttB	7884	94.9	38.2
23	113419	+	mttB	7037	95.0	35.7
23	113536	+	mttB	10774	77.7	38.1
23	113564	+	mttB	11805	96.3	38.6
23	113654	+	mttB	6393	96.5	37.6
23	113662	+	mttB	5107	94.3	37.2
23	113767	+	mttB	14684	97.5	37.5
23	113870	+	mttB	10718	98.5	37.3
24	36129	+	rpl5-pseudo	756	21.6	37.0
24	36438	+	rpl5-pseudo	554	53.4	37.9
24	36614	+	rpl5-pseudo	1131	52.3	38.0
25	70948	-	cox3	46307	98.4	36.4
25	71370	-	UTR-cox3-atp8	47824	37.2	38.1
25	71684	-	atp8	42085	43.3	37.7
25	72109	-	atp8	15728	96.5	36.7
26	88065	+	IGS	547	78.2	38.9
26	88474	+	IGS	435	33.1	36.7
26	88698	+	IGS	628	67.7	37.1
28	12812	-	nad7-4	82769	99.8	38.5
28	12875	-	nad7-4	44787	99.6	37.3
28	12890	-	nad7-4	42614	99.7	37.8
28	13015	-	nad7-4	24931	97.8	37.2
28	13448	-	intron-nad7	5285	29.8	38.1
28	13533	-	intron-nad7	3941	31.9	35.2
28	14025	-	intron-nad7	3283	84.4	36.9
28	15243	-	nad7-3	22851	98.4	34.8
28	15274	-	nad7-3	16346	99.1	37.3
28	15570	-	nad7-3	22927	98.9	34.5
28	17404	-	nad7-1	11965	93.6	38.3
28	17443	-	nad7-1	18289	92.2	36.2
28	17659	-	5UTR-nad7	16619	51.4	38.9
30	35023	+	nad2-pseudo	529	22.1	39.1
30	35677	+	nad2-pseudo	8419	98.2	37.5
30	35682	+	nad2-pseudo	8373	98.3	37.1
30	35715	+	nad2-pseudo	9679	97.0	35.6
30	35749	+	nad2-pseudo	9024	98.2	38.9
30	35818	+	nad2-pseudo	488	78.7	39.1
32	53108	-	matR-a	11802	87.2	36.8
32	53149	-	matR-a	9387	22.1	37.2
32	53234	-	matR-a	7492	99.1	36.9
32	53755	-	matR-a	4574	60.1	38.0
32	54058	-	matR-a	3155	23.9	36.1
32	54653	-	matR-a	2628	96.0	37.6

32	54786	-	matR-a	13973	99.1	36.5
32	54947	-	matR-a	5453	61.6	36.8
32	55672	-	nad1-4	1957	23.6	34.2
32	56121	-	intron/IGS-nad1-4	2865	30.0	37.8
32	56239	-	intron/IGS-nad1-4	3390	52.1	36.0
33	71	-	IGS	742	39.0	38.2
33	29863	-	IGS	645	40.2	39.7
33	107948	-	IGS	396	25.3	38.3
33	108157	-	IGS	620	20.7	36.8
33	108431	-	IGS	638	24.9	36.4
35	36945	-	nad1-1	16693	85.9	38.0
35	36987	-	nad1-1	14316	98.3	36.8
35	37250	-	nad1-1	14939	66.2	38.3
35	37404	-	5UTR-nad1-1	16994	31.3	36.2
36	33354	+	nad3-a	6388	71.3	37.6
36	33355	+	nad3-a	6388	23.0	37.3
36	33501	+	nad3-a	5325	95.4	38.2
36	33508	+	nad3-a	5207	89.3	38.7
36	33642	+	nad3-a	7891	95.5	39.4
38	66220	-	atp6-pseudo	2038	76.4	38.6
38	66274	-	atp6-pseudo	38730	99.7	39.6
38	66411	-	atp6-pseudo	9151	99.8	36.5
38	66475	-	atp6-pseudo	6720	99.1	37.8
38	66478	-	atp6-pseudo	6746	99.4	38.1
38	66537	-	atp6-pseudo	10741	99.4	37.2
38	66667	-	IGS	12000	99.6	38.6
38	67482	-	IGS	11566	84.3	37.0
38	67559	-	IGS	5923	95.3	38.8
38	67574	-	IGS	5998	95.3	37.1
39	49370	-	atp6	112502	99.8	39.4
39	49424	-	atp6	137427	99.0	36.8
39	49561	-	atp6	54413	99.8	38.3
39	49625	-	atp6	115223	99.5	38.3
39	49628	-	atp6	115192	99.7	38.5
39	49972	-	atp6	95611	99.6	37.9
39	50051	-	atp6	52075	98.9	35.7
39	50849	-	atp1	38708	30.6	36.6
41	30934	+	IGS	5152	56.5	37.1
41	31045	+	IGS	3813	83.8	37.7
42	33886	+	5UTR-rps13	3483	22.5	38.6
42	34519	+	5UTR-rps13	19914	29.7	37.9
42	35465	+	3UTR-rps13	330	39.4	37.8
42	35856	+	3UTR-rps13	474	71.3	38.0

42	35871	+	3UTR-rps13	463	70.0	37.6
42	35934	+	3UTR-rps13	339	54.0	36.3
45	94414	-	nad2-pseudo	836	99.0	33.3
45	94534	-	nad2-pseudo	22249	99.8	39.3
45	94603	-	nad2-pseudo	10371	99.0	34.2
46	19783	-	IGS	1722	33.1	38.7
46	45056	+	5UTR-ccmFn	615	21.5	37.3
46	45240	+	ccmFn	1114	97.3	38.5
46	45285	+	ccmFn	589	91.7	35.4
46	45443	+	ccmFn	951	98.1	37.2
46	45666	+	ccmFn	1626	41.2	36.8
46	45843	+	ccmFn	1677	98.0	36.6
46	45912	+	ccmFn	936	99.4	36.5
46	45939	+	ccmFn	439	99.5	33.4
46	46134	+	ccmFn	1255	21.7	37.5
46	46409	+	ccmFn	780	99.6	38.4
46	46437	+	ccmFn	660	98.8	36.8
46	46454	+	ccmFn	547	98.5	35.7
46	46469	+	ccmFn	679	98.5	35.3
46	46487	+	ccmFn	829	98.9	37.6
46	46520	+	ccmFn	1146	95.2	38.0
46	46531	+	ccmFn	1249	26.3	37.1
46	46538	+	ccmFn	1223	98.5	38.3
46	46712	+	ccmFn	1651	70.6	37.3
47	48627	-	rps3	14592	98.9	38.0
47	48654	-	rps3	9329	98.3	36.6
47	49248	-	rps3	6181	26.8	35.1
47	49296	-	rps3	4139	97.3	36.8
47	51315	-	5UTR-rps3	1236	68.3	38.3
47	52264	-	IGS	1212	39.7	38.4
47	52595	-	IGS	289	39.5	38.0
49	31208	+	nad2-3	24657	31.0	37.6
49	33112	+	nad2-4	42441	99.5	38.7
49	33142	+	nad2-4	45968	99.5	38.7
49	33146	+	nad2-4	50364	98.9	38.1
49	33212	+	nad2-4	28309	90.7	38.4
49	33242	+	nad2-4	34939	99.4	36.1
49	33311	+	nad2-4	24403	99.8	38.9
49	33431	+	nad2-4	3982	82.9	37.0
49	34782	+	nad2-5	154508	98.5	36.2
49	34785	+	nad2-5	160482	99.0	35.2
49	34790	+	nad2-5	155520	96.2	35.5
54	3422	+	UTR-rrn5	202	62.9	37.2

54	3492	+	UTR-rrn5	248	50.8	36.0
55	1642	-	nad3-b	7667	97.3	39.4
55	1776	-	nad3-b	4872	89.3	38.8
55	1783	-	nad3-b	5028	95.5	38.3
55	1929	-	nad3-b	6352	23.7	37.3
55	1930	-	nad3-b	6352	71.0	37.7
55	70100	-	cox2	34143	99.2	37.5
55	70192	-	cox2	27276	98.7	37.0
55	71541	-	5UTR-cox2	4273	79.9	38.2
57	60684	-	atp6-pseudo	1574	98.3	38.6
57	60738	-	atp6-pseudo	38878	99.7	39.6
57	60875	-	atp6-pseudo	9119	99.7	36.5
57	60939	-	atp6-pseudo	6550	99.1	37.7
57	60942	-	atp6-pseudo	6568	99.5	38.1
57	61001	-	atp6-pseudo	10809	99.4	37.3
57	61131	-	IGS	12088	99.6	38.6
57	61946	-	IGS	11426	84.2	37.0
57	62023	-	IGS	5956	95.6	38.7
57	62038	-	IGS	5996	95.6	37.1
60	66673	-	3UTR-atp4-a	30489	50.7	37.8
60	67379	-	atp4	25399	99.4	36.5
60	67535	-	atp4	12301	99.6	37.7
60	67559	-	atp4	15773	99.6	36.7
60	67571	-	atp4	18399	99.7	37.4
60	67697	-	atp4	14692	99.7	38.4
60	67752	-	atp4	11772	81.3	36.8
60	67944	-	nad4L-a	8746	98.9	38.2
60	68095	-	nad4L-a	9427	99.4	38.1
60	68116	-	nad4L-a	8532	98.9	38.0
60	68131	-	nad4L-a	8840	99.5	38.5
60	68179	-	nad4L-a	6910	99.6	39.1
60	68224	-	nad4L-a	3494	89.0	38.2
60	69653	-	UTR-nad4L-a-ccmB-b	4237	89.3	37.5
60	69933	-	UTR-nad4L-a-ccmB-b	6464	81.0	37.2
60	69962	-	UTR-nad4L-a-ccmB-b	6276	79.1	37.8
60	70427	-	ccmB-b	131	84.7	36.8
60	70436	-	ccmB-b	295	96.6	35.7
60	70463	-	ccmB-b	726	96.3	38.9
60	70626	-	ccmB-b	647	98.9	38.2
60	70766	-	ccmB-b	180	88.3	33.6
60	70770	-	ccmB-b	130	83.1	34.0
60	70781	-	ccmB-b	382	100.0	37.4
60	70859	-	ccmB-b	274	92.3	38.8

60	70860	-	ccmB-b	274	92.3	38.8
60	70902	-	ccmB-b	482	97.7	38.0
60	71821	-	5UTR-ccmB-b	310	94.2	37.3
60	72095	-	5UTR-ccmB-b	295	50.2	37.5
61	4373	-	matR-b	11881	86.8	36.8
61	4414	-	matR-b	9532	22.1	37.4
61	4499	-	matR-b	7509	98.9	36.9
61	5020	-	matR-b	4464	60.9	37.8
61	5323	-	matR-b	3161	23.0	36.2
61	5918	-	matR-b	2719	96.1	37.8
61	6051	-	matR-b	13778	99.2	36.4
61	6212	-	matR-b	5399	63.6	36.6
61	6937	-	nad1-4	1887	24.5	34.5
61	7386	-	intron/IGS-nad1-4	2917	30.6	37.5
61	7504	-	intron/IGS-nad1-4	3348	52.2	36.1
62	43183	-	3UTR-atp4-b	30577	50.6	37.8
62	43889	-	atp4-b	25461	99.3	36.5
62	44045	-	atp4-b	12091	99.6	37.7
62	44069	-	atp4-b	15741	99.7	36.7
62	44081	-	atp4-b	18443	99.8	37.4
62	44207	-	atp4-b	14643	99.8	38.4
62	44262	-	atp4-b	11757	80.6	36.8
62	44454	-	nad4L-b	8678	98.9	38.1
62	44605	-	nad4L-b	9326	99.3	38.1
62	44626	-	nad4L-b	8478	99.2	38.0
62	44641	-	nad4L-b	8794	99.6	38.5
62	44689	-	nad4L-b	6912	99.6	39.2
62	44734	-	nad4L-b	3544	88.3	38.2
62	46163	-	UTR-nad4L-b-ccmB-c	4431	89.4	37.5
62	46443	-	UTR-nad4L-b-ccmB-c	6381	80.2	37.1
62	46472	-	UTR-nad4L-b-ccmB-c	6127	78.4	37.8
62	46937	-	ccmB-c	147	73.5	37.0
62	46946	-	ccmB-c	308	95.5	35.6
62	46973	-	ccmB-c	751	95.1	38.8
62	47136	-	ccmB-c	579	98.6	38.3
62	47276	-	ccmB-c	202	92.6	33.7
62	47280	-	ccmB-c	151	88.1	33.7
62	47286	-	ccmB-c	119	91.6	34.6
62	47369	-	ccmB-c	283	90.1	38.5
62	47370	-	ccmB-c	283	90.1	38.5
62	47412	-	ccmB-c	500	98.2	37.9

Table S5. Summary of non-canonical (i.e., other C-to-U) editing sites identified by the CLC algorithm. Sites labeled as Intron/IGS were found adjacent to an exon with a *trans*-spliced intron with unknown boundaries, so it is not clear whether they are in the intron or IGS. UTRs and IGSs were defined/distinguished based on an arbitrary cutoff of 2 kb from annotated coding sequences.

Chrom	Position	Strand	Gene/Location	Reference	Alternative	Coverage	Edited (%)	Mean Quality
1	132088	+	3UTR-cob	T	A	4327	27.34	32.01
8	93669	+	5UTR-nad2	A	C	252	41.67	32.77
11	95250	+	Intron-nad4	G	C	1137	55.50	11.70
21	23158	+	Intron/IGS-nad5-exon3	T	C	4171	59.89	38.43
21	23177	+	Intron/IGS-nad5-exon3	G	A	3791	56.98	38.97
21	23220	+	Intron/IGS-nad5-exon3	A	T	3896	53.85	36.18
33	29936	-	IGS	A	G	599	31.89	34.12
33	29937	-	IGS	T	C	599	31.89	34.12
36	33691	+	3UTR-nad3	T	A	652	20.09	31.76
41	31418	+	IGS	T	C	4137	59.71	38.39
41	31437	+	IGS	G	A	3766	56.72	38.73
41	31480	+	IGS	A	T	3808	52.76	35.93
47	48342	-	3UTR-rps3	A	T	1393	36.47	32.29
55	1593	-	3UTR-nad3	A	T	308	37.66	31.22

Table S6. Comparison of identified RNA editing sites in protein-coding genes to previous analysis of RNA editing in another *Silene noctiflora* population by Sloan et al. (2010)

Chrom	Position	Strand	Gene[a]	Coverage	Edited (%)	Mean Quality	2010 Study
1	128225	+	ccmB	706	99.43	37.47	Y
1	128267	+	ccmB	564	96.81	38.22	Y
1	128268	+	ccmB	564	96.81	38.22	Y
1	128351	+	ccmB	165	97.58	34.52	Y
1	128357	+	ccmB	212	88.68	33.59	Y
1	128361	+	ccmB	262	95.80	33.51	Y
1	128501	+	ccmB	523	99.43	38.01	Y
1	128664	+	ccmB	898	97.22	39.00	Y
1	128691	+	ccmB	550	97.82	35.72	Y
1	128700	+	ccmB	269	82.90	36.77	Y
1	129165	+	rpl5	9124	91.98	37.80	Y
1	129194	+	rpl5	9213	92.79	37.72	Y
1	129474	+	rpl5	3879	94.10	37.45	Y
1	129660	+	rpl5	27632	90.88	37.09	Y
1	131199	+	cob	31293	98.68	38.23	Y
1	131232	+	cob	19165	98.89	35.69	Y
1	131442	+	cob	26100	99.49	39.05	Y
1	131682	+	cob	8374	98.88	34.89	Y
1	131782	+	cob	35816	98.30	37.57	Y
1	132034	+	cob	126110	99.76	39.22	Y
2	111595	-	ccmFc	6047	99.67	38.14	Y
2	111669	-	ccmFc	8243	98.11	36.99	Y
2	113335	-	ccmFc	3976	99.57	36.29	Y
2	113598	-	ccmFc	3832	91.73	37.40	Y
2	113602	-	ccmFc	3745	99.23	36.97	Y
2	113630	-	ccmFc	2659	87.89	38.15	Y
2	113703	-	ccmFc	3529	98.07	35.43	Y
3	87856	+	nad1-3	10633	95.60	36.20	Y
3	87866	+	nad1-3	15401	89.76	36.19	Y
3	87902	+	nad1-3	30676	94.51	36.47	Y
4	85458	+	nad5-1	10696	97.09	36.64	Y
4	86410	+	nad5-2	19807	98.91	37.30	Y
4	86542	+	nad5-2	24318	99.41	38.29	Y
4	86566	+	nad5-2	33424	99.48	36.64	Y
4	86716	+	nad5-2	21415	97.48	36.83	Y
4	86721	+	nad5-2	23496	96.87	36.55	Y
4	86776	+	nad5-2	26054	99.52	38.23	Y
4	86893	+	nad5-2	30369	98.75	33.81	Y
4	87003	+	nad5-2	37968	99.59	35.59	Y
4	87478	+	nad5-2	65369	99.62	36.17	Y
6	76528	+	atp9	226140	99.00	35.90	Y
6	144066	-	nad1-5	53836	99.24	36.74	Y

6	144096	-	nad1-5	30260	99.22	37.14	Y
6	144251	-	nad1-5	10431	97.95	34.86	Y
7	38551	+	nad5-4	54499	97.68	36.78	Y
7	38581	+	nad5-4	92780	99.44	36.18	Y
7	38611	+	nad5-4	71512	99.68	37.71	Y
7	39920	+	nad5-5	41655	98.84	37.03	Y
7	39983	+	nad5-5	69836	99.41	37.24	Y
8	94380	+	nad2-1	29476	93.73	37.92	Y
8	95686	+	nad2-2	8556	98.14	37.51	Y
8	95691	+	nad2-2	8508	98.19	37.12	Y
8	95724	+	nad2-2	9708	96.82	35.61	Y
8	95758	+	nad2-2	22091	98.37	36.32	Y
8	95827	+	nad2-2	23403	97.83	39.04	Y
8	112014	+	nad6	41731	90.94	36.82	Y
8	112015	+	nad6	41731	90.94	36.82	Y
8	112021	+	nad6	40101	85.62	36.44	Y
8	112072	+	nad6	27070	99.60	38.48	Y
8	112117	+	nad6	12981	99.32	36.95	Y
8	112495	+	nad6	38230	99.81	38.61	Y
9	111015	-	ccmC	14144	99.77	39.24	Y
9	111038	-	ccmC	14765	99.35	38.50	Y
9	111074	-	ccmC	8482	97.74	36.52	Y
9	111112	-	ccmC	7311	58.92	39.03	Y
9	111189	-	ccmC	4161	97.38	37.07	Y
9	111214	-	ccmC	3255	28.60	37.95	N
9	111215	-	ccmC	3255	70.08	37.56	Y
9	111221	-	ccmC	3284	98.29	37.71	Y
9	111225	-	ccmC	3896	98.64	37.48	Y
9	111230	-	ccmC	4074	98.50	37.64	Y
9	111237	-	ccmC	3815	98.32	38.26	Y
9	111242	-	ccmC	3725	99.25	38.49	Y
9	111293	-	ccmC	5003	98.96	37.34	Y
9	111357	-	ccmC	3271	98.14	36.05	Y
9	111504	-	ccmC	3977	98.42	37.34	Y
9	111585	-	ccmC	4296	91.29	38.00	Y
10	65731	+	nad9	21147	98.95	35.61	Y
11	94270	+	nad4-1	11376	96.83	38.28	Y
11	94303	+	nad4-1	16521	95.87	37.15	Y
11	94354	+	nad4-1	17231	96.36	37.61	Y
11	94393	+	nad4-1	15488	98.55	38.11	Y
11	94564	+	nad4-1	11174	98.06	38.21	Y
11	94629	+	nad4-1	6029	87.23	37.76	Y
11	94633	+	nad4-1	5933	97.39	36.59	Y
11	96760	+	nad4-2	16081	98.25	36.57	Y
11	97105	+	nad4-2	5321	98.74	39.10	Y
11	98436	+	nad4-3	29316	98.96	37.12	Y

11	98452	+	nad4-3	29492	99.22	39.18	Y
23	113221	+	mttB	4634	92.43	37.82	Y
23	113257	+	mttB	6540	91.45	38.01	Y
23	113288	+	mttB	7884	94.91	38.24	Y
23	113419	+	mttB	7037	95.01	35.73	Y
23	113536	+	mttB	10774	77.70	38.06	Y
23	113564	+	mttB	11805	96.31	38.55	Y
23	113654	+	mttB	6393	96.45	37.60	Y
23	113662	+	mttB	5107	94.34	37.18	Y
23	113767	+	mttB	14684	97.49	37.50	Y
23	113870	+	mttB	10718	98.49	37.32	Y
25	70948	-	cox3	46307	98.40	36.40	Y
25	71684	-	atp8	42085	43.32	37.69	Y
25	72109	-	atp8	15728	96.52	36.72	Y
28	12812	-	nad7-4	82769	99.83	38.53	Y
28	12875	-	nad7-4	44787	99.56	37.33	Y
28	12890	-	nad7-4	42614	99.69	37.78	Y
28	13015	-	nad7-4	24931	97.83	37.18	Y
28	15243	-	nad7-3	22851	98.38	34.82	Y
28	15274	-	nad7-3	16346	99.06	37.27	Y
28	15570	-	nad7-3	22927	98.92	34.49	Y
28	17404	-	nad7-1	11965	93.64	38.32	Y
28	17443	-	nad7-1	18289	92.21	36.20	Y
32	53108	-	matR-a	11802	87.17	36.84	Y
32	53149	-	matR-a	9387	22.13	37.23	N
32	53234	-	matR-a	7492	99.05	36.89	Y
32	53755	-	matR-a	4574	60.14	38.04	Y
32	54058	-	matR-a	3155	23.87	36.12	N
32	54653	-	matR-a	2628	96.00	37.55	Y
32	54786	-	matR-a	13973	99.11	36.49	Y
32	54947	-	matR-a	5453	61.64	36.78	Y
35	36945	-	nad1-1	16693	85.86	38.00	Y
35	36987	-	nad1-1	14316	98.29	36.77	Y
35	37250	-	nad1-1	14939	66.19	38.33	Y
36	33354	+	nad3-a	6388	71.29	37.61	Y
36	33355	+	nad3-a	6388	22.95	37.34	Y
36	33501	+	nad3-a	5325	95.38	38.19	Y
36	33508	+	nad3-a	5207	89.32	38.71	Y
36	33642	+	nad3-a	7891	95.46	39.37	Y
39	49370	-	atp6	112502	99.83	39.42	Y
39	49424	-	atp6	137427	99.00	36.83	Y
39	49561	-	atp6	54413	99.77	38.34	Y
39	49625	-	atp6	115223	99.52	38.26	Y
39	49628	-	atp6	115192	99.69	38.54	Y
39	49972	-	atp6	95611	99.62	37.93	Y
39	50051	-	atp6	52075	98.85	35.73	Y

39	50849	-	atp1	38708	30.59	36.55	N
46	45240	+	ccmFn	1114	97.31	38.45	Y
46	45285	+	ccmFn	589	91.68	35.43	Y
46	45443	+	ccmFn	951	98.11	37.23	Y
46	45666	+	ccmFn	1626	41.21	36.84	N
46	45843	+	ccmFn	1677	98.03	36.64	Y
46	45912	+	ccmFn	936	99.36	36.50	Y
46	45939	+	ccmFn	439	99.54	33.42	Y
46	46134	+	ccmFn	1255	21.67	37.45	N
46	46409	+	ccmFn	780	99.62	38.37	Y
46	46437	+	ccmFn	660	98.79	36.80	Y
46	46454	+	ccmFn	547	98.54	35.70	Y
46	46469	+	ccmFn	679	98.53	35.29	Y
46	46487	+	ccmFn	829	98.91	37.59	Y
46	46520	+	ccmFn	1146	95.20	37.95	Y
46	46531	+	ccmFn	1249	26.26	37.11	Y
46	46538	+	ccmFn	1223	98.45	38.29	Y
46	46712	+	ccmFn	1651	70.62	37.31	Y
47	48627	-	rps3	14592	98.94	37.98	Y
47	48654	-	rps3	9329	98.33	36.55	Y
47	49248	-	rps3	6181	26.84	35.14	N
47	49296	-	rps3	4139	97.32	36.82	Y
49	31208	+	nad2-3	24657	30.99	37.59	Y
49	33112	+	nad2-4	42441	99.53	38.69	Y
49	33142	+	nad2-4	45968	99.50	38.66	Y
49	33146	+	nad2-4	50364	98.86	38.12	Y
49	33212	+	nad2-4	28309	90.66	38.43	Y
49	33242	+	nad2-4	34939	99.40	36.06	Y
49	33311	+	nad2-4	24403	99.82	38.86	Y
49	33431	+	nad2-4	3982	82.92	37.04	Y
49	34782	+	nad2-5	154508	98.54	36.19	Y
49	34785	+	nad2-5	160482	99.02	35.19	Y
49	34790	+	nad2-5	155520	96.19	35.51	Y
55	70100	-	cox2	34143	99.22	37.52	Y
55	70192	-	cox2	27276	98.65	37.04	Y
60	67379	-	atp4-a	25399	99.41	36.51	Y
60	67535	-	atp4-a	12301	99.57	37.72	Y
60	67559	-	atp4-a	15773	99.61	36.72	Y
60	67571	-	atp4-a	18399	99.70	37.43	Y
60	67697	-	atp4-a	14692	99.72	38.35	Y
60	67752	-	atp4-a	11772	81.30	36.81	Y
60	67944	-	nad4L-a	8746	98.94	38.17	Y
60	68095	-	nad4L-a	9427	99.41	38.12	Y
60	68116	-	nad4L-a	8532	98.85	37.96	Y
60	68131	-	nad4L-a	8840	99.50	38.45	Y
60	68179	-	nad4L-a	6910	99.57	39.14	Y

60	68224	-	nad4L-a	3494	88.95	38.21	Y
61	6937	-	nad1-4	1887	24.54	34.45	Y

[a]Only a single representative is shown for genes with multiple copies in the genome.

Table S7. Summary of RNA editing sites that were previously reported in another *Silene noctiflora* population by Sloan et al. (2010) but not identified by the CLC algorithm at a cutoff of 20% editing frequency

Chrom	Position	Strand	Gene	Manual Inspection[a]
1	128208	+	ccmB	Not Edited
1	128325	+	ccmB	Highly Edited
1	128345	+	ccmB	Highly Edited
1	128346	+	ccmB	Highly Edited
1	128711	+	ccmB	Highly Edited
1	128748	+	ccmB	Highly Edited
1	128751	+	ccmB	Highly Edited
1	128769	+	ccmB	Highly Edited
1	128793	+	ccmB	Highly Edited
2	113631	-	ccmFc	Highly Edited
23	131163	+	mttB	Partially Edited
35	36944	-	nad1-1	Highly Edited
46	46583	+	ccmFn	Not Edited
49	33396	+	nad2-4	Partially Edited

[a]Manual inspection of all mapped RNA-seq reads was used to classify each site: "Highly Edited" indicates >80%; "Partially Edited" indicates <20%; and "Not Edited" indicates no evidence of editing.
